# Synthesis of new bile acid-fused tetrazoles using the Schmidt reaction

**DOI:** 10.3762/bjoc.17.174

**Published:** 2021-10-20

**Authors:** Dušan Đ Škorić, Olivera R Klisurić, Dimitar S Jakimov, Marija N Sakač, János J Csanádi

**Affiliations:** 1 Department of Chemistry, Biochemistry and Environmental Protection, Faculty of Sciences, University of Novi Sad, Trg Dositeja Obradovića 3, 21000 Novi Sad, Serbia; 2 Department of Physics, Faculty of Sciences, University of Novi Sad, Trg Dositeja Obradovića 4, 21000 Novi Sad, Serbia; 3 Oncology Institute of Vojvodina, Faculty of Medicine, University of Novi Sad, Put Dr Goldmana 4, 21204 Sremska Kamenica, Serbia

**Keywords:** antiproliferative activity, Schmidt reaction, steroids, tetrazoles, trimethylsilyl azide

## Abstract

A practical and high-yielding Schmidt reaction for the synthesis of fused tetrazoles from bile acid precursors was developed. Mild reaction conditions using TMSN_3_ instead of hydrazoic acid as an azide source produced good yields of the desired tetrazoles. These conditions could be applied to other steroidal precursors. Additionally, an improved methodology for the synthesis of different ketone and enone precursors from cholic acid, deoxycholic acid, and chenodeoxycholic acid was established. Newly obtained tetrazole derivatives were characterized by NMR and X-ray diffraction spectroscopy. In a number of cases, preliminary antiproliferative tests of new compounds showed strong and selective activity towards certain tumor cell lines.

## Introduction

Bile acids are naturally occurring steroidal surfactants that play various biological roles. Besides the well-known role as lipid solubilizers, bile acids are now recognized as metabolism regulators through specific receptors: farnesoid X receptor (FXR) and Takeda G protein receptor 5 (TGR5) [1–3]. Research efforts to find ligands for these receptors initiated several synthetic studies where bile acids are being used as a starting material [4–7]. Aggregation and interactions with the cell membrane of bile acid molecules can alter drug action [8–9]. Our previous work showed that bile acids could interact with certain drug molecules and improve their biological activity [10–11]. To evade the membranolytic action of natural bile acids, derivatives with altered hydrophobicity are being studied [12–14].

**Figure 1:**
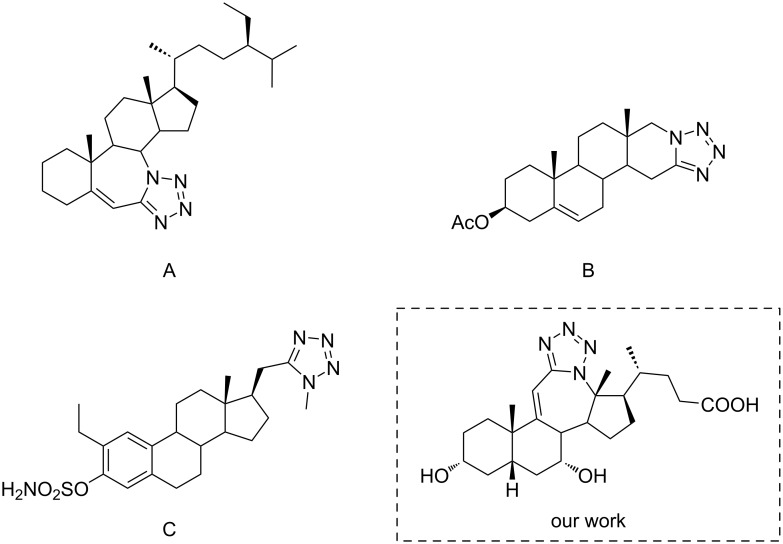
Structures of the steroidal tetrazoles that showed anticancer potential in vitro.

The tetrazole moiety can be found in many biologically active compounds, and monosubstituted tetrazole is being used in medicinal chemistry as a bioisostere of carboxylic acid [15] because it increases the lipophilicity and metabolic stability of the molecule [16]. Steroid molecules with nitrogen-containing heterocyclic rings are promising candidates for the treatment of many types of cancer or hormonal disorders [17]. There are several examples of steroidal tetrazoles showing anticancer potential (Figure 1) [18–19].

**Figure 2:**
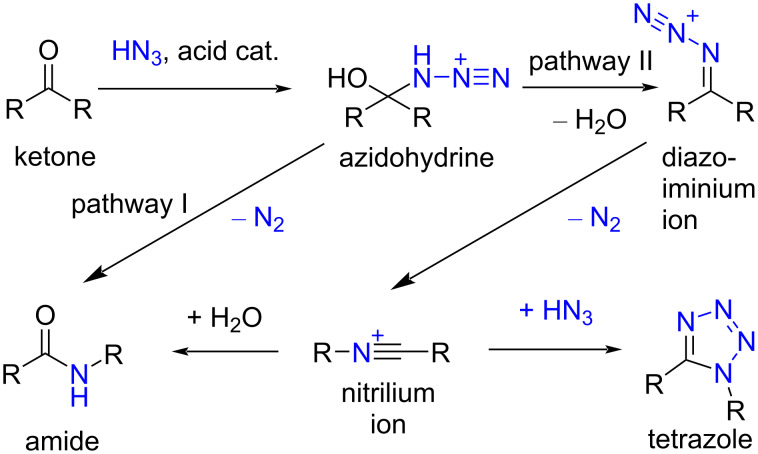
Mechanism of the Schmidt reaction.

The main approach in the synthesis of the tetrazoles is 1,3-dipolar cycloaddition between azide and nitrile. These reactions often follow the principles of “click” chemistry [20]. Although the formation of tetrazole in the Schmidt reaction of ketones was noted in the original study by Schmidt himself [21], this variation of the reaction draws considerably less attention in comparison to the usage in the synthesis of amides or lactams. As presented in Figure 2, after initial formation of the azidohydrine by addition of hydrazoic acid to the ketone, the reaction can undergo two pathways. In the first pathway, the amide product is formed exclusively, while in the second pathway, the elimination of water from the azidohydrine affords a diazoiminium ion, which rearranges to a nitrilium ion by eliminating a nitrogen molecule. The addition of water to this nitrilium ion gives the amide product, but if the excess of hydrazoic acid is present, tetrazole can be formed as an alternative product. It is established that the use of nonaqueous conditions and concentrated mineral acids or Lewis acids favors the second pathway [22], and thus increasing the possibility of tetrazole formation. Intramolecular Schmidt reaction of alkyl azides and ketones, which follows the first pathway in the mechanism, found especially broad application in the synthesis of different lactams [23–24]. Serious drawbacks of the Schmidt reaction for tetrazole synthesis are the need for a large excess of the hazardous hydrazoic acid and the formation of lactam, which often prevails, especially when hydrazoic acid is generated in situ by the action of Brønsted acid on sodium azide [25]. The use of trimethylsilyl azide (TMSN_3_) instead of hydrazoic acid for many transformations has gained attention since TMSN_3_ is less hazardous [26–27]. Some recent studies employ this reagent in the Schmidt synthesis of tetrazoles from ketones that are smaller in size and simpler than the steroidal ketones in this work [28–29].

For the synthesis of steroidal tetrazoles, most often 1,3-dipolar cycloadditions are being used. In this way, derivatives of bile acid, androstene, and cholestane were prepared, with the tetrazole ring not being fused to the steroid core [30–33]. Some fused steroidal tetrazole derivatives were obtained by intramolecular 1,3-dipolar cycloaddition [34–35]. It should be noted that the Schmidt reaction, employing hydrazoic acid, was used in transformations of some steroidal ketones to the corresponding lactams and tetrazoles. Often in these cases, the yield of the tetrazole was low, and the steroids used as starting material lacked potentially reactive functional groups [36–39].

Referring to the above-mentioned potential of a nitrogen-containing steroids, and as a continuation of our research in the field of bile acids and steroidal heterocycles, in this work, we aimed to prepare a series of new bile acid tetrazoles with potential cytotoxicity towards selected tumor cells. To achieve the synthesis of these compounds, we have been working to establish reliable protocol for the Schmidt synthesis of fused tetrazoles from bile acid ketones and enones that would have potential for application in the synthesis of other steroidal molecules. New B-ring- and C-ring-fused steroidal tetrazoles obtained in this way were subjected to antiproliferative activity testing in vitro. It is worth mentioning that the fused tetrazole derivatives of bile acids may possess better hydrophobic–hydrophilic balance, important for aggregation properties, which could be an interesting topic for future research.

## Results and Discussion

**Scheme 1:**
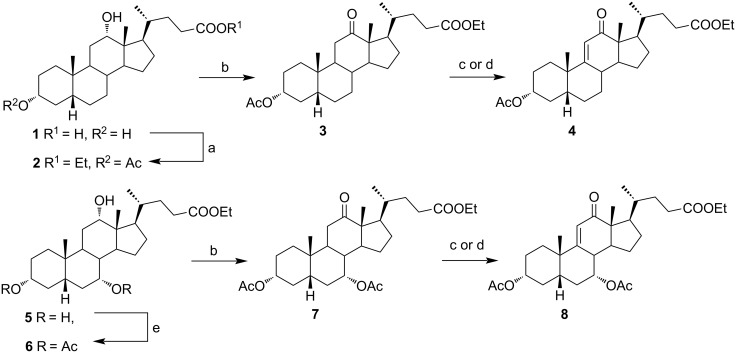
Synthesis of 12-oxo intermediates. Reagents and conditions: a) EtOAc, pTsOH, reflux, 14 h (81%); b) K_2_Cr_2_O_7_, H_3_O^+^, H_2_O/Et_2_O, rt, 3 h (76% for **3**; 71% for **7**); c) SeO_2_, acetic acid, reflux, 12 h (74% for **4**; 69% for **8**); d) SeO_2_, acetic acid, microwave irradiation, 150 °C, 15 min (74% for **4**; 67% for **8**); and e) Ac_2_O, benzene/pyridine, rt, 24 h (74%).

**Scheme 2:**
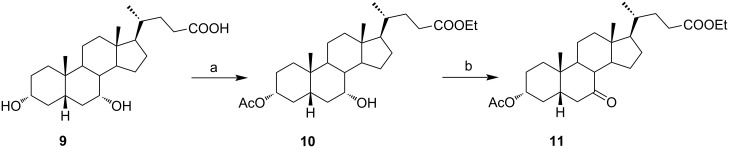
Synthesis of 7-oxo intermediate **11** from chenodeoxycholic acid (**9**). Reagents and conditions: a) EtOAc, pTsOH, reflux, 12 h (66%) and b) K_2_Cr_2_O_7_, H_3_O^+^, H_2_O/Et_2_O, rt, 3 h (84%).

In order to investigate possibilities for the synthesis of bile acid B-ring- and bile acid C-ring-fused tetrazoles by a variation of the Schmidt reaction, the appropriate ketone and enone derivatives were prepared firstly. This was achieved using cholic acid, deoxycholic acid (**1**), and chenodeoxycholic acid (**9**), respectively, as starting material. Ethyl cholate (**5**) and 7-oxo derivative **12** were prepared from cholic acid following well-known procedures [40–41]. The synthesis of other oxo compounds is outlined in Scheme 1 and Scheme 2. Deoxycholic acid (**1**) and chenodeoxycholic acid (**9**) were selectively esterified in refluxing ethyl acetate with a catalytic amount of *p*-toluenesulfonic acid (pTsOH) [42]. Subsequent oxidation of the free OH groups afforded compounds **3** and **11** in high yields. Similarly, compound **7** was prepared in good overall yield from ethyl cholate (**5**) by selective acetylation [43], followed by oxidation. Enones **4** and **8** were prepared by dehydrogenation of corresponding ketones with SeO_2_ in refluxing acetic acid [44]. Microwave-assisted heating of the reaction mixture in a closed vessel (150 °C) helped in decreasing the reaction time for dehydrogenation noticeably, while the yield was preserved.

Ketone **3** and enone **4** were used for the optimization of reaction conditions. Firstly, the ketone **3** was reacted with hydrazoic acid in the presence of boron trifluoride etherate as a Lewis acid (Table 1, entry 1). The desired tetrazole **13** was obtained after chromatographic purification. A lactam byproduct was detected in a small quantity, the isolation and purification of which proved to be problematic. Further work was directed towards reactions using less hazardous TMSN_3_ as an azide source. Data for entries 2–8 in Table 1 are showing that trimethylsilyl trifluoromethanesulfonate (TMSOTf) is superior to BF_3_⋅OEt_2_ as a catalyst in both dichloromethane (DCM) and acetonitrile (ACN), while ACN appears to be the better choice as solvent. A particularly good yield was obtained with TMSOTf in ACN. Also, it is evident that an increase in the amount of TMSN_3_ and Lewis acid did not provide any significant change in yield. Myers and co-workers described BF_2_OTf⋅OEt_2_ as a powerful Lewis acid formed in situ from BF_3_⋅OEt_2_ and TMSOTf, which was especially efficient in ACN [45]. This prompted us to investigate the application of BF_2_OTf⋅OEt_2_ in our synthesis (Table 1, entries 9–11). As we expected, the reaction in ACN was the most efficient, while in DCM, there were no significant differences between BF_2_OTf⋅OEt_2_ and TMSOTf. Lowering reaction temperature in ACN (Table 1, entry 10) increased the yield of the tetrazole **13**. Again, an increased amount of TMSN_3_ and catalyst did not provide any significant improvement in the yield. In all cases where TMSN_3_ was used as an azide source, the lactam byproduct was not detected.

**Table 1:** Optimization of reaction conditions for the transformation of ketones.

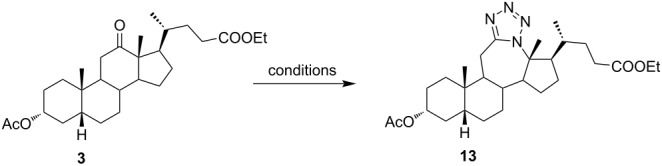				

entry	conditions	equiv of azide/equiv of Lewis acid	reaction time	yield (%)^a^

1^b^	HN_3_, BF_3_·OEt_2_, benzene, rt	16:5	48 h	58
2	TMSN_3_, TMSOTf, DCM, rt	6:3	24 h	70
3	TMSN_3_, TMSOTf, DCM, rt	12:6	24 h	70
4	TMSN_3_, TMSOTf, ACN, rt	6:3	3 h	82
5	TMSN_3_, BF_3_·OEt_2_, DCM, rt	6:3	48 h	62
6	TMSN_3_, BF_3_·OEt_2_, DCM, rt	12:6	48 h	63
7	TMSN_3_, BF_3_·OEt_2_, ACN, rt	6:3	24 h	71
8	TMSN_3_, BF_3_·OEt_2_, ACN, rt	12:6	24 h	70
9^c^	TMSN_3_, BF_3_·OEt_2_, TMSOTf, ACN, rt	6:3	4 h	71
10^c^	TMSN_3_, BF_3_·OEt_2_, TMSOTf, ACN, 0–5 °C	6:3	6 h	76
11^c^	TMSN_3_, BF_3_·OEt_2_, TMSOTf, DCM, rt	12:6	24 h	72

^a^Isolated yield. ^b^Concentration of hydrazoic acid solution obtained by the literature protocol that we used is between 6 and 10% [36]. ^c^Equimolar amounts of Lewis acids were added to the reaction mixture in sequence.

Of special interest for us was the synthesis of conjugated tetrazoles from enones. Since the oxygen atom in the enone form has a lower affinity towards Lewis acid, the reactivity of these compounds in the Schmidt reaction is lower. It is known that different conjugated ketones require a longer time to react and give lower yields [28,46–47]. Furthermore, enones can react in both 1,2- and 1,4-fashion, increasing the possibility for side reactions. Reddy and co-workers reported Lewis acid-mediated enone reactions with alkyl azides yielding enaminones [48]. Similarly, as with ketone, reactivity of the enone derivative **4** with HN_3_ in the presence of BF_3_⋅OEt_2_ (Table 2, entry 1) was examined. It was shown that a longer time is needed to achieve conversion, and the yield was lower in comparison to ketone. Again, the corresponding lactam was identified in low yield, but the isolation of the compound was not accomplished. A reaction in DCM, catalyzed by TMSOTf, appeared to be optimal for the synthesis of conjugated tetrazoles in 69% yield. It was shown that the reaction in ACN, although having fast conversion, yields a complex mixture of products with a low isolated yield of the desired tetrazole. It could be observed that a longer reaction time was needed for conversions of enone, and that the reaction performance was largely unchanged by the increase of azide and Lewis acid concentration, or by the decrease of temperature.

**Table 2:** Optimization of reaction conditions for the transformation of enones.

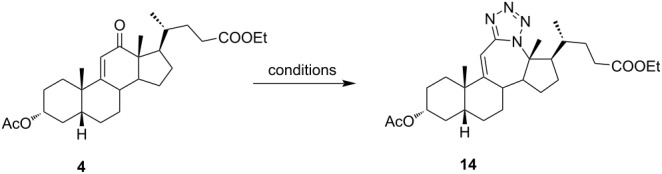				

entry	conditions	equiv of azide/equiv of Lewis acid	reaction time	yield (%)^a^

1^b^	HN_3_, BF_3_·OEt_2_, benzene, rt	16:5	72 h	52
2	TMSN_3_, TMSOTf, DCM, rt	6:3	72 h	69
3	TMSN_3_, TMSOTf, DCM, rt	12:6	48 h	64
4	TMSN_3_, TMSOTf, ACN, rt	6:3	4 h	42
5	TMSN_3_, BF_3_·OEt_2_, DCM, rt	6:3	72 h	66
6	TMSN_3_, BF_3_·OEt_2_, DCM, rt	12:6	72 h	68
7	TMSN_3_, BF_3_·OEt_2_, ACN, rt	6:3	48 h	51
8	TMSN_3_, BF_3_·OEt_2_, ACN, rt	12:6	48 h	51
9^c^	TMSN_3_, BF_3_·OEt_2_, TMSOTf, ACN, rt	6:3	4 h	40
10^c^	TMSN_3_, BF_3_·OEt_2_, TMSOTf, ACN, 0–5 °C	6:3	4 h	42
11^c^	TMSN_3_, BF_3_·OEt_2_, TMSOTf, DCM, rt	12:6	24 h	42

^a^Isolated yield. ^b^Concentration of hydrazoic acid solution obtained by the literature protocol that we used is between 6 and 10% [36]. ^c^Equimolar amounts of Lewis acids were added to the reaction mixture in sequence.

**Figure 3:**
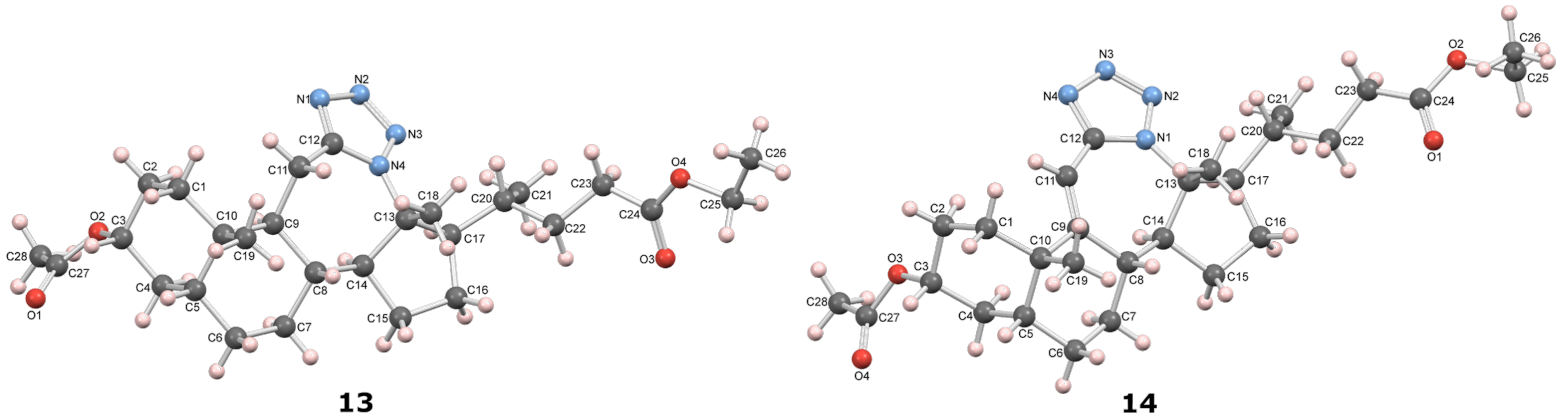
Mercury [51] drawing of the molecular structures of compounds **13** and **14**, with labelling of nonhydrogen atoms. Hydrogen atoms are drawn as spheres of arbitrary radii.

The use of TMSN_3_ enabled a more efficient exclusion of water from the reaction mixture in comparison to a HN_3_ solution in benzene, which is difficult to dry thoroughly. This, in turn, was helpful in shifting the Schmidt reaction towards tetrazole formation. In support of this was the fact that no lactam byproduct was detected in reactions carried out with TMSN_3_. As it was confirmed by detailed NMR analysis (see Supporting Information File 1), only homoregioisomeric 12a-azatetrazoles were obtained. This is in good correlation with earlier observations about the preferred migration of the more substituted carbon atom during the Schmidt reaction [49]. Also, a retention of configuration at the C-13 position was confirmed by NOE NMR experiments. The chemical shift of some protons in compounds **13** and **14** exhibited effects of magnetic anisotropy from the aromatic tetrazole ring. A characteristic example of this is the H-17 proton in compound **13** with an unusually high chemical shift (2.85 ppm). Unambiguous confirmation of the tetrazole molecular structure came from the XRD analysis of compounds **13** and **14** (Figure 3) [50–51].

The already established and optimized reaction conditions were tested on a 1 mmol scale with different bile acid ketones and enones (Table 3). For ketones, ACN was used as reaction solvent, while for enone derivatives, DCM was applied. Desired tetrazoles were obtained in good to excellent yields. 7-Oxo derivatives appeared to be less reactive in comparison to the 12-oxo counterparts, which can be attributed to the fact that the migrating carbon atom (C-8) is less substituted than the C-13 carbon atom. Structures of all compounds were determined by a detailed NMR analysis. For B-homotetrazoles **17** and **18,** a retention of configuration at C-8 was confirmed. Similarly to C-homotetrazoles, magnetic anisotropy effects of the aromatic tetrazole ring are obvious in ^1^H NMR spectra of compounds **17** and **18**. For example, the chemical shift of the H-4α proton is much lower than expected (0.45 ppm). Interestingly, we noticed that crystals of protected B-homotetrazoles were not obtained after repeated attempts of crystallization, while obtaining high-quality crystals of C-homotetrazoles was straightforward. In the final part of our synthetic work, all tetrazole compounds were converted to free bile acid-fused tetrazoles with ethanolic KOH solution at room temperature in high yields.

**Table 3:** Synthesis of different B-ring-fused and C-ring-fused bile acid tetrazoles.^a^

starting compound^b^	product of Schmidt reaction	product of total deprotection

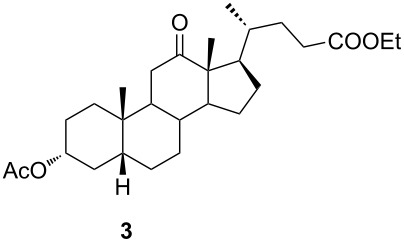	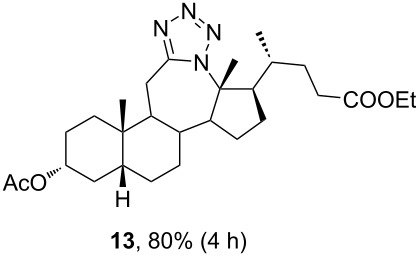	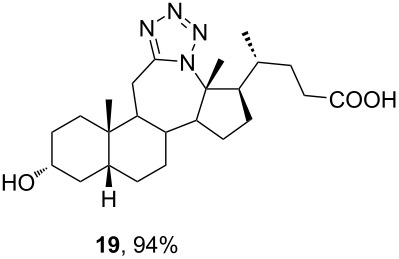
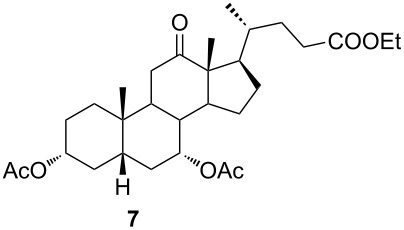	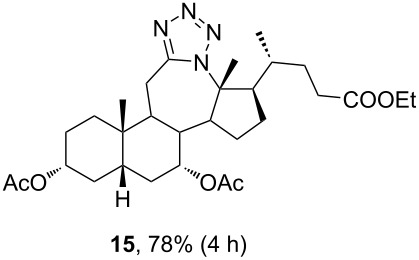	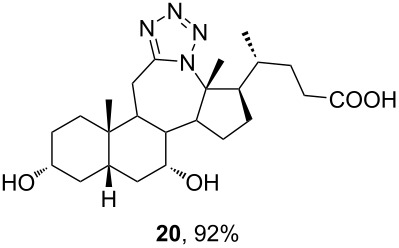
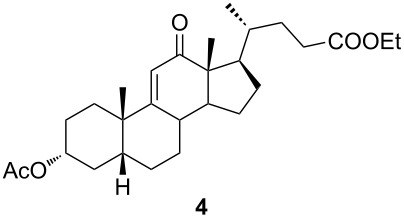	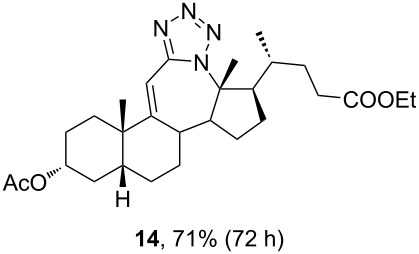	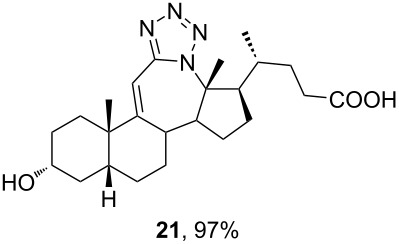
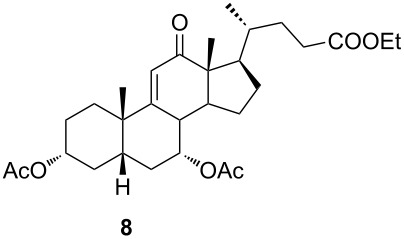	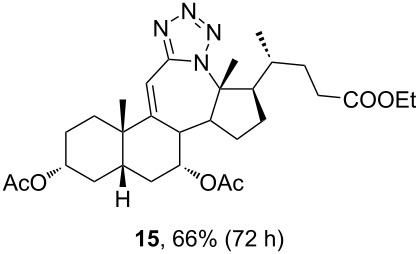	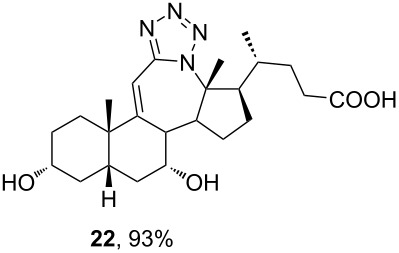
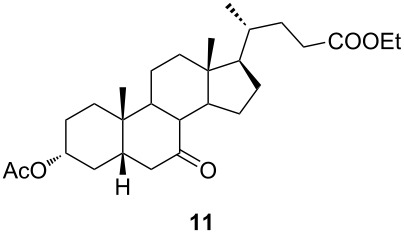	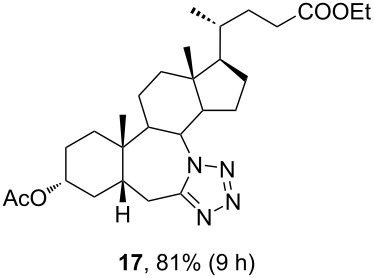	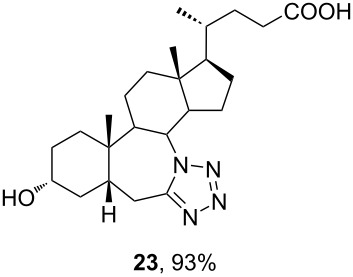
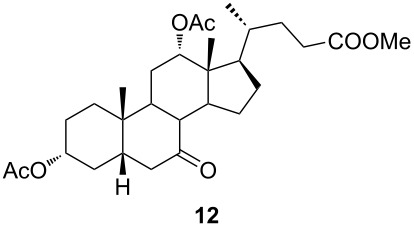	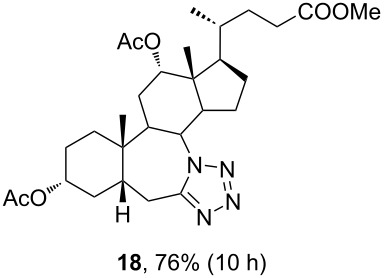	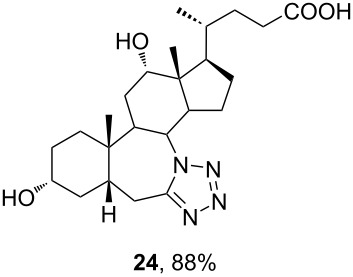

^a^Synthesis of tetrazoles was performed according to previously optimized conditions on a 1 mmol scale with 6 equiv of TMSN_3_ and 3 equiv of TMSOTf. For ketones, ACN was used as a solvent, while for enones, DCM was the reaction solvent. ^b^Compound **12** was prepared according to a literature procedure [41].

In order to gain basic information on the biological activity of synthesized compounds, in vitro antiproliferative activity of compounds **3**, **4**, **7**, **8**, and **11**–**24** was determined. One normal human cell line (MRC-5 fetal fibroblasts) was used together with six human tumor cell lines (MCF-7 estrogen receptor positive breast adenocarcinoma, MDA-MB-231 estrogen receptor negative breast adenocarcinoma, PC-3 prostate cancer, HeLa cervix carcinoma, HT-29 colon adenocarcinoma, and A549 adenocarcinomic human alveolar basal epithelial cells). Standard MTT assay [52] was used with commercial nonselective antitumor agent doxorubicin (DOX) as control [53]. The results of antiproliferative in vitro analysis of all tested compounds are shown in Table 4.

**Table 4:** Results of antiproliferative activity testing (IC_50_ values less than 10 μM are marked with an asterisk, while a dash denotes an IC_50_ value higher than 100 μM).

compound	IC_50_ (μM), 72 h, MTT test						

	MCF-7	MDA-MB-231	PC-3	HeLa	HT-29	A549	MRC-5

**3**	—	1.06*	78.55	8.68*	—	—	N/A^a^
**4**	14.42	70.64	9.60*	—	—	17.73	N/A
**7**	—	9.18*	—	—	—	—	—
**8**	—	—	43.11	—	—	4.25*	N/A
**11**	—	96.31	26.29	18.16	—	—	N/A
**12**	—	82.42	11.19	43.71	—	36.57	—
**13**	—	—	—	69.61	16.39	—	—
**14**	—	—	—	6.97*	24.07	—	—
**15**	—	—	—	21.65	23.59	—	N/A
**16**	—	98.90	33.90	43.04	68.07	—	—
**17**	—	—	—	95.91	—	19.56	—
**18**	22.92	22.46	82.60	19.48	27.15	79.75	—
**19**	—	14.79	—	12.07	34.35	—	N/A
**20**	N/A	87.47	—	—	61.74	—	—
**21**	37.24	21.67	—	9.31*	98.50	—	N/A
**22**	—	45.57	—	—	6.06*	—	—
**23**	—	7.60*	11.44	—	—	—	N/A
**24**	—	5.18*	12.26	77.61	—	—	—
DOX [53]	0.20*	0.09*	84.23	0.07*	0.15*	4.91*	0.10*

^a^IC_50_ value not available due to nonlinear dose dependence or hormetic effect.

**Figure 4:**
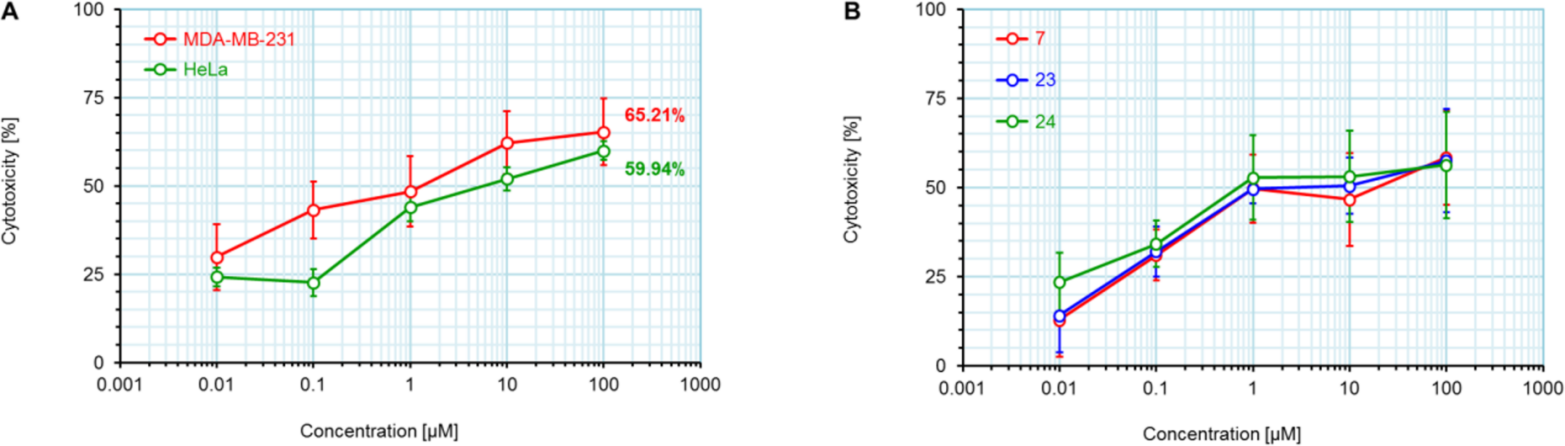
Dose dependence of the cytotoxicity of tested compounds on treated cell lines. All compounds were tested in the concentration range 0.1–100 μM. A) Cytotoxicity of compound **3** against MDA-MB-231 and HeLa cell lines. B) Cytotoxicity of compounds **7**, **23**, and **24** on breast cancer cell line MDA-MB-231.

Among all tested compounds, ketone **3** showed the lowest IC_50_ value (1.06 μM) towards MDA-MB-231 cells, with linear dose dependence of cytotoxicity through the tested concentration range (Figure 4A). Compound **3** also showed strong activity on the HeLa cell line. Introduction of tetrazole ring instead of ketone diminished activity toward the MDA-MB-231 cell line in compound **13** (IC_50_ > 100 μM). The same trend with MDA-MB-231 cells was noticeable with compounds **7** and **15**. Interestingly, the introduction of tetrazole into the B-ring of cholic acid scaffold increased activity toward MDA-MB-231 cells dramatically (IC_50_ of **12** = 82.42 μM vs IC_50_ of **24** = 5.18 μM). Further, tetrazole **14** showed strong and selective activity toward the HeLa cell line (IC_50_ = 6.97 μM), while tetrazole **22** showed strong and selective activity toward the HT-29 cell line (IC_50_ = 6.06 µM). Compounds **7**, **23**, and **24**, which showed strong cytotoxicity to the breast cancer cell line MDA-MB-231 (in addition to compound **3**), also exhibited a mutually very similar mode of action. Looking at the cytotoxicity dose dependence of **7**, **23**, and **24** on MDA-MB-231 cell line (Figure 4B), it could be seen that it is not completely linear. At a concentration greater than 1 µM, cytotoxicity did not increase at the same rate but a plateau-like curve was formed. This means that a higher concentration is not as effective and that a better effect is achieved at a lower concentration of the compound, which is a good feature for an antitumor drug candidate. None of the tested compounds exhibited toxicity toward the normal cell line MRC-5.

## Conclusion

In this work, modified and optimized reaction conditions for the Schmidt synthesis were established for the preparation of bile acid-fused tetrazoles using TMSN_3_. It was shown that depending on the starting compound, ACN was optimal for ketones and DCM was optimal for enones. Among the tested Lewis acid catalysts, TMSOTf proved to be the most efficient. High yields of the desired tetrazole compounds with no lactam byproduct were obtained. The molecular structure and stereochemistry of newly synthesized tetrazoles was established by detailed NMR analysis. For compounds **13** and **14**, the structure was established by additional XRD analysis. A preliminary test of the antiproliferative activity showed that the introduction of C-ring-fused tetrazole lowered the activity towards some tumor cell lines compared to the corresponding ketone, while B-ring-fused tetrazole increased these activities.

## Experimental

### General procedure for the preparation of tetrazoles with hydrazoic acid

Caution: Hydrazoic acid should be handled with care in an efficient fume hood since it is toxic and explosive.

In a two-neck round-bottom flask equipped with an addition funnel and argon inlet, a benzene solution of hydrazoic acid (18 mL) and boron trifluoride etherate (1.15 mL) were placed. The mixture was cooled with an ice water bath, and a benzene solution of the carbonyl compound (1.50 mmol in 15 mL of benzene) was added dropwise over 40 minutes with stirring and cooling. After the addition was completed, the reaction mixture was left at room temperature with continuous stirring until the end of the reaction. After reaction completion, the mixture was poured into water, extracted with ethyl acetate, and the combined organic extracts were washed with saturated NaHCO_3_ solution and water. After drying and evaporation of the solvent in vacuo, crude products were obtained and were purified by column chromatography.

### General procedure for preparation of tetrazoles with TMSN_3_

In a flame-dried two-neck round-bottom flask equipped with an argon inlet, 0.10 mmol of the carbonyl compound was dissolved in 4 mL of dry solvent, and 6–12 equiv of TMSN_3_ and 3–6 equiv of Lewis acid were added through the septum. The reaction mixture was stirred under an inert atmosphere (see Table 1 and Table 2). After reaction completion, the mixture was poured into the water (60 mL), extracted with ethyl acetate, and the combined organic extracts were washed with saturated NaHCO_3_ solution and water. After drying and evaporation of the solvent in vacuo, crude products were obtained and were purified by column chromatography.

### General procedure for deprotection

In a round-bottom flask, the protected bile acid tetrazole (0.20 mmol) was dissolved in an ethanolic KOH solution (10 mL, 5% w/v). The reaction mixture was stirred at room temperature for 24 h. After reaction completion the mixture was concentrated in vacuo and poured into water. Then, the pH value was adjusted to ≈3 with 1 M HCl. The mixture was extracted with ethyl acetate, and the combined organic extracts were washed with water. After drying and evaporation of the solvent in vacuo, the obtained crude products were crystallized from the appropriate solvent.

## Supporting Information

File 1 Synthetic procedures, analytical data, X-ray analysis details, and copies of spectra.

## References

[1] Stofan M, Guo G L (2020). Front Med.

[2] Sepe V, Distrutti E, Limongelli V, Fiorucci S, Zampella A (2015). Future Med Chem.

[3] Pols T W H, Noriega L G, Nomura M, Auwerx J, Schoonjans K (2011). Dig Dis.

[4] Pellicciari R, Passeri D, De Franco F, Mostarda S, Filipponi P, Colliva C, Gadaleta R M, Franco P, Carotti A, Macchiarulo A (2016). J Med Chem.

[5] Festa C, Renga B, D’Amore C, Sepe V, Finamore C, De Marino S, Carino A, Cipriani S, Monti M C, Zampella A (2014). J Med Chem.

[6] Macchiarulo A, Gioiello A, Thomas C, Pols T W H, Nuti R, Ferrari C, Giacchè N, De Franco F, Pruzanski M, Auwerx J (2013). ACS Med Chem Lett.

[7] Gioiello A, Venturoni F, Tamimi S, Custodi C, Pellicciari R, Macchiarulo A (2014). Med Chem Commun.

[8] Faustino C, Serafim C, Rijo P, Pinto Reis C (2016). Expert Opin Drug Delivery.

[9] Darkoh C, Lichtenberger L M, Ajami N, Dial E J, Jiang Z-D, DuPont H L (2010). Antimicrob Agents Chemother.

[10] Poša M, Csanádi J, Kövér K E, Guzsvány V, Batta G (2012). Colloids Surf, B.

[11] Aleksić Sabo V, Škorić D, Jovanović-Šanta S, Nikolić I, János C, Knežević P (2021). J Ethnopharmacol.

[12] Bjedov S, Jakimov D, Pilipović A, Poša M, Sakač M (2017). Steroids.

[13] Bjedov S, Jakimov D, Poša M, Klisurić O R, Sakač M (2017). Tetrahedron.

[14] Poša M, Bjedov S, Škorić D, Sakač M (2015). Biochim Biophys Acta, Gen Subj.

[15] Ostrovskii V A, Trifonov R E, Popova E A (2012). Russ Chem Bull.

[16] Herr R J (2002). Bioorg Med Chem.

[17] Bansal R, Acharya P C (2014). Chem Rev.

[18] Jourdan F, Bubert C, Leese M P, Smith A, Ferrandis E, Regis-Lydi S, Newman S P, Purohit A, Reed M J, Potter B V L (2008). Org Biomol Chem.

[19] Zhang J, Wang S, Ba Y, Xu Z (2019). Eur J Med Chem.

[20] Demko Z P, Sharpless K B (2002). Angew Chem, Int Ed.

[21] Schmidt K F (1924). Ber Dtsch Chem Ges B.

[22] Amyes T L, Richard J P (1991). J Am Chem Soc.

[23] Gu P, Kang X-Y, Sun J, Wang B-J, Yi M, Li X-Q, Xue P, Li R (2012). Org Lett.

[24] Li X-J, Qiao J-B, Sun J, Li X-Q, Gu P (2014). Org Lett.

[25] Cervantes A, Crabbe P, Iriarte J, Rosenkranz G (1968). J Org Chem.

[26] Hajra S, Bhowmick M, Sinha D (2006). J Org Chem.

[27] Omura M, Iwanami K, Oriyama T (2007). Chem Lett.

[28] Motiwala H F, Charaschanya M, Day V W, Aubé J (2016). J Org Chem.

[29] Li L-H, Niu Z-J, Li Y-X, Liang Y-M (2018). Chem Commun.

[30] Stoltz K L, Erickson R, Staley C, Weingarden A R, Romens E, Steer C J, Khoruts A, Sadowsky M J, Dosa P I (2017). J Med Chem.

[31] Bukiya A N, Patil S A, Li W, Miller D D, Dopico A M (2012). ChemMedChem.

[32] Kádár Z, Kovács D, Frank É, Schneider G, Huber J, Zupkó I, Bartók T, Wölfling J (2011). Molecules.

[33] Uzzaman S, Asif M, Ali A, Mashrai A, Khanam H, Sherwani A, Owais M (2014). Eur Chem Bull.

[34] Gogoi J, Bezbaruah P, Saikia P, Goswami J, Gogoi P, Boruah R C (2012). Tetrahedron Lett.

[35] Penov-Gaši K M, Oklješa A M, Petri E T, Ćelić A S, Djurendić E A, Klisurić O R, Csanadi J J, Batta G, Nikolić A R, Jakimov D S (2013). Med Chem Commun.

[36] Moural J, Syhora K (1970). Collect Czech Chem Commun.

[37] Ahmad M S, Chaudhry Z H, Khan P N (1976). Aust J Chem.

[38] Dwivedy I, Singh A K, Singh M M, Ray S (1993). Steroids.

[39] Alam M, Nami S A A, Husain A, Lee D-U, Park S (2013). C R Chim.

[40] Rasras A J M, Al-Tel T H, Al-Aboudi A F, Al-Qawasmeh R A (2010). Eur J Med Chem.

[41] Iuliano A, Facchetti S, Uccello-Barretta G (2006). J Org Chem.

[42] Kuhajda K, Kandrač J, Cirin-Novta V, Miljković D (1996). Collect Czech Chem Commun.

[43] Fieser L F, Rajagopalan S (1950). J Am Chem Soc.

[44] Fieser L F, Rajagopalan S, Wilson E, Tishler M (1951). J Am Chem Soc.

[45] Myers E L, Butts C P, Aggarwal V K (2006). Chem Commun.

[46] Litkei G, Patonay T (1983). Acta Chim Hung.

[47] Mphahlele M J (2009). Trends Org Chem.

[48] Reddy D S, Judd W R, Aubé J (2003). Org Lett.

[49] Bach R D, Wolber G J (1982). J Org Chem.

[R50] 50CCDC 2102193–2102194 contain the supplementary crystallographic data for this paper. These data can be obtained free of charge via https://www.ccdc.cam.ac.uk/data_request/cif or by emailing data_request@ccdc.cam.ac.uk or by contacting The Cambridge Crystallographic Data Centre, 12 Union Road, Cambridge CB2 1EZ, U.K. Fax: +441223336033.

[51] Macrae C F, Sovago I, Cottrell S J, Galek P T A, McCabe P, Pidcock E, Platings M, Shields G P, Stevens J S, Towler M (2020). J Appl Crystallogr.

[52] Mosmann T (1983). J Immunol Methods.

[53] Jakimov D S, Kojić V V, Aleksić L D, Bogdanović G M, Ajduković J J, Djurendić E A, Penov Gaši K M, Sakač M N, Jovanović-Šanta S S (2015). Bioorg Med Chem.

