# Supplementary material for: Synthesis of new bile acid-fused tetrazoles using the Schmidt reaction
Source: Beilstein J Org Chem. 2021 Oct 20;17:2611–20. doi: 10.3762/bjoc.17.174 (PMC8551880; doi:10.3762/bjoc.17.174)
Supplement: File 1 — Synthetic procedures, analytical data, X-ray analysis details, and copies of spectra. [file Beilstein_J_Org_Chem-17-2611-s001.pdf]

BEILSTEIN JOURNAL OF ORGANIC CHEMISTRY

Supporting Information

for

Synthesis of new bile acid-fused tetrazoles using the Schmidt reaction

Dušan Đ. Škorić, Olivera R. Klisurić, Dimitar S. Jakimov, Marija N. Sakač
and János J. Csanádi

Beilstein J. Org. Chem. **2021,** *17,* 2611–2620. doi:10.3762/bjoc.17.174

Synthetic procedures, analytical data, X-ray analysis details, and copies of spectra

License and Terms: This is a supporting information file under the terms of the Creative Commons Attribution License (https://creativecommons.org/licenses/by/4.0). Please note that the reuse, redistribution and reproduction in particular requires that the author(s) and source are credited and that individual graphics may be subject to special legal provisions.
The license is subject to the *Beilstein Journal of Organic Chemistry* terms and conditions: (https://www.beilstein-journals.org/bjoc/terms)

Content

1. **Experimental procedures and analytical data…………………………… S1**
2. **X-ray crystallography data………………………………………………….. S15**
3. **^{1}H and ^{13}C NMR spectra…………………………………………….............. S17**
4. **References…………………………………………………………………… S34**

1. Experimental procedures and analytical data

General information

All reactions requiring dry conditions were conducted in flame-dried glassware under an inert atmosphere of argon. Acetonitrile was dried over molecular sieves and redistilled prior to use, DCM and benzene were distilled over P_2O_5, and pyridine was dried over KOH and redistilled prior to use. All organic solutions were dried with anhydrous Na_2SO_4. TLC was carried out on Merck 60 F_{245} aluminium-backed silica gel plates, UV light or 50% sulfuric acid with heating were used for visualization. Chromatographic purifications were performed using silica gel (0,04–0,063 mm, Merck). NMR spectra were recorded on Bruker AC 250 (250 MHz for ^{1}H, 62 MHz for ^{13}C) and Bruker AV III HD 400 (400 MHz for ^{1}H and 101 MHz for ^{13}C) instruments. Residual solvent signals were used for spectra calibrations. For microwave-assisted reactions, CEM Discovery Benchmate reactor was used. IR spectra were recorded on a PerkinElmer SpectrumTwo instrument. High-resolution mass spectroscopy (HRMS) was recorded on an Agilent Technologies 6210 (ESI+) LC–MS (time-of-flight) instrument or a Thermo LTQ OrbiTrap instrument. Melting points were determined on a hot-stage apparatus and are uncorrected.

Ethyl 3α-acetoxy-12α-hydroxy-5β-cholan-24-oate (2)

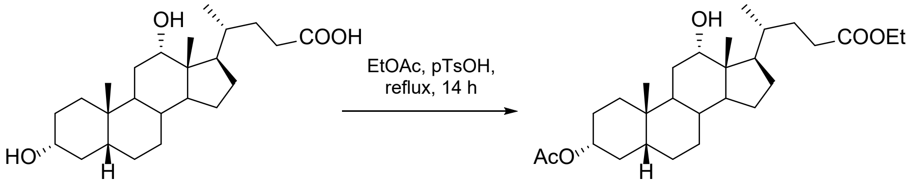

1 **2**

Deoxycholic acid (**1**, 2.00 g, 5.09 mmol) was dissolved in ethyl acetate (50 mL) in a round-bottom flask. *p*-Toluenesulfonic acid (0.200 g, 1.16 mmol) and water (0.6 mL) were added, and the reaction mixture was refluxed for 14 h. After reaction completion, the mixture was poured into saturated $NaHCO_3$ solution (150 mL), the phases were

separated, and the water phase was extracted with ethyl acetate (3 × 50 mL). Combined organic extracts were washed with water, dried, and the solvent was evaporated in vacuo, giving the crude product, which was purified by column chromatography (toluene/acetone 6:1, R_f: 0.35). Yield: 81% (1.91 g), colorless oil. Spectroscopic data are in good correlation with the literature data [1].

Ethyl 3α-acetoxy-12-oxo-5β-cholan-24-oate (3)

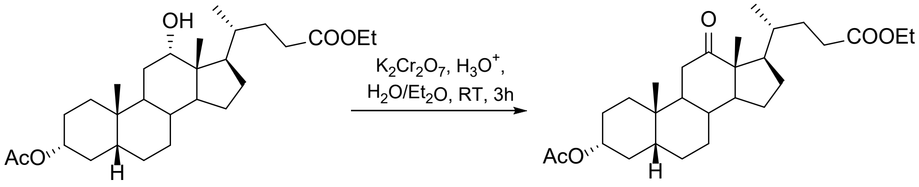

2 **3**

Compound **2** (1.06 g, 2.29 mmol) was dissolved in diethyl ether (20 mL). An acidified solution of $K_2Cr_2O_7$ (1.01 g in 10 mL H_2O) was added dropwise with vigorous stirring. The reaction mixture was kept at room temperature for 3 h with vigorous stirring. After reaction completion, the mixture was transferred to the separation funnel, and the phases were separated. The water phase was extracted with diethyl ether (3 × 20 mL), and the combined organic phases were washed with saturated $NaHCO_3$ solution and water. After drying and evaporation of the solvent in vacuo, the product was obtained and was used further without additional purification. Yield: 76% (0.81 g, 1.74 mmol), pale yellow amorphous crystals.

R_f: 0.82 (toluene/acetone 4:1) IR (KBr) $\tilde{\nu}$ = 2958, 2878, 1715 cm^{-1}.

^1H NMR (250 MHz, $CDCl_3$): 0.84 (d, J=6.4 Hz, 3H, H-21,); 1.00 (s, 6H, H-19, H-18); 4.11 (q, J=7.4 Hz, 2H, -O-**CH_2**CH_3); 4.68 (m, 1H, H-3).

^{13}C NMR (62 MHz, $CDCl_3$): 11.6 (CH_3); 14.2 (CH_3); 18.5 (CH_3); 21.3 (CH_3); 22.7 (CH_3); 24.3 (CH_2); 25.9 (CH_2); 26.3 (CH_2); 26.9 (CH_2); 27.4 (CH_2); 30.4 (CH_2); 31.5 (CH_2); 32.1 (CH_2); 34.8 (CH_2); 35.3 (Cq); 35.5 (CH); 35.6 (CH); 38.0 (CH_2); 41.3 (CH); 43.9 (CH); 46.4 (CH); 57.4 (Cq); 58.6 (CH); 60.1 (CH_2); 73.6 (CH, C-3); 170.5 (Cq, AcO); 174.1 (Cq, C-24); 214.5 (Cq, C-12).

Ethyl 3α-acetoxy-12-oxo-5β-chol-9(11)-en-24-oate (4)

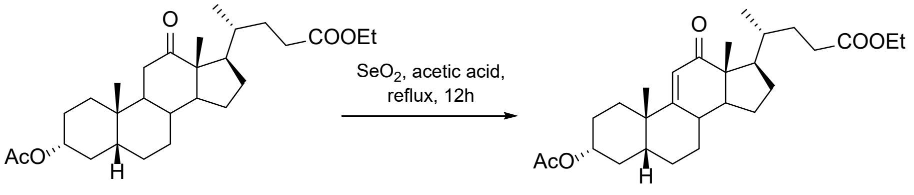

3 4

Compound **3** (0.923 g, 2.00 mmol) was dissolved in glacial acetic acid (20 mL), SeO_2 (0.750 g, 6.76 mmol) was added, and the reaction mixture was refluxed for 12 h. After reaction completion, the mixture was diluted with 50 mL of DCM and filtered through the pad of Celite®. Further, the mixture was washed with saturated $NaHCO_3$ solution and water. After drying and evaporation of the solvent in vacuo, the crude product was obtained and purified by column chromatography (petroleum ether/acetone 12:1, R_f:0.23). Yield: 74% (0.678 g), white crystals from acetone/hexane, mp 148 °C.

IR (KBr) ṽ = 2934, 2870, 1731, 1673 cm^{-1}.

^1H NMR (400 MHz, $CDCl_3$): 0.91 (s, 3H, H-18); 1.02 (d, J=6.5 Hz, 3H, H-21); 1.19 (s, 3H, H-19); 1.25 (t, J=7.2 Hz, 3H, -O-CH_2**CH_3**,); 2.00 (s, 3H, **CH_3**CO-); 4.12 (q, J=7.2 Hz, 2H, -O-**CH_2**CH_3); 4.73 (m, 1H, H-3); 5.71 (d, J=2.2 Hz, 1H, H-11).

^{13}C NMR (101 MHz, $CDCl_3$): 10.74; 14.27; 19.50; 21.37; 24.23; 26.20; 26.54; 27.41; 27.67; 29.77; 30.66; 31.78; 34.03; 35.06; 35.31; 37.85; 39.96; 41.84; 47.31; 53.08; 53.53; 60.18; 73.81 (CH, C-3); 123.71 (CH, C-11); 163.99 (Cq, C-9); 170.64 (Cq, CH_3CO-); 174.29 (Cq, C-24); 205.13 (Cq, C-12).

Ethyl 3α,7α-diacetoxy-12α-hydroxy-5β-cholan-24-oate (6)

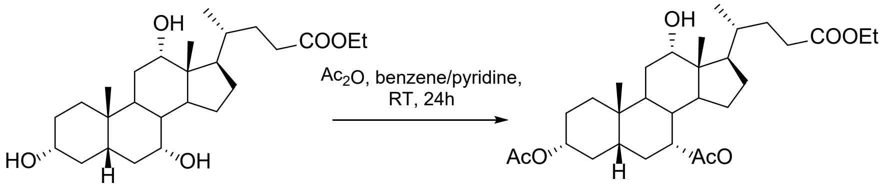

ethyl-cholate 6

Ethyl cholate (**5**, 5.09 g, 11.52 mmol) was dissolved in dry benzene (60 mL) and dry pyridine (7.5 mL), and acetic anhydride (7.5 mL) was added. The reaction mixture was kept at room temperature with stirring for 24 h. After reaction completion, the mixture was poured into water (250 mL) and the phases were separated. The water phase was extracted with ethyl acetate (3 × 40 mL), and the combined organic phases were

washed with 1 M HCl and water. After drying and evaporation of the solvent in vacuo, te crude product was obtained and was crystallized from methanol. The product was used further without additional purification. Yield: 74% (4.43 g).

Ethyl 3α,7α-diacetoxy-12-oxo-5β-cholan-24-oate (7)

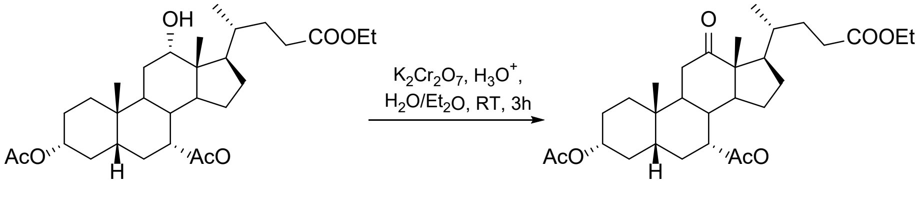

Compound **6** (2.00 g, 3.85 mmol) was dissolved in diethyl ether (50 mL). An acidified solution of $K_2Cr_2O_7$ (2.10 g in 20 mL H_2O) was added dropwise with vigorous stirring. The reaction mixture was kept at room temperature for 3 h with vigorous stirring. After reaction completion, the mixture was transferred to the separation funnel and the phases were separated. The water phase was extracted with diethyl ether (3 × 20 mL), and the combined organic phases were washed with saturated $NaHCO_3$ solution and water. After drying and evaporation of the solvent in vacuo, the product was obtained and purified by column chromatography (toluene/acetone 7:1, R_f: 0.40). Yield 71% (1.41 g), colorless crystals from ethanol/hexane, mp 163 °C.

IR (KBr) ṽ = 2935, 2862, 1711 cm^{-1}.

^{1}H NMR (400 MHz, $CDCl_3$): 0.85 (d, J=6.5 Hz, 3H, H-21); 1.02 (s, 6H, H-19, H-18); 1.24 (t, J=7.1 Hz, 3H, -O-CH_2**CH_3**,); 2.01-2.02 (s, 6H, 2 × **CH_3**CO-); 4.11 (q, J=7.1 Hz, 2H, -O-**CH_2**CH_3); 4.56 (m, 1H, H-3); 4.98 (d, J=2.7 Hz, 1H, H-7).

^{13}C NMR (101 MHz, $CDCl_3$): 11.49; 14.26; 18.54; 21.42; 21.46; 22.10; 23.78; 26.53; 27.37; 30.44; 31.32; 31.49; 34.52; 34.87; 35.50; 35.53; 37.60; 37.84 (2 × CH); 40.46; 46.34; 53.11; 57.07; 60.19; 70.48; 73.51; 170.16 (Cq, CH_3CO-); 170.65 (Cq, CH_3CO-); 174.15 (Cq, C-24); 213.92 (Cq, C-12).

Ethyl 3α,7α-diacetoxy-12-oxo-5β-chol-9(11)-en-24-oate (8)

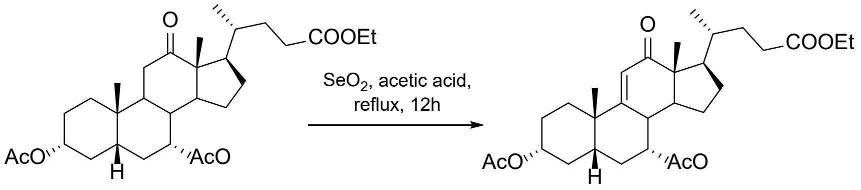

7 **8**

Compound **7** (0.400 g, 0.77 mmol) was dissolved in glacial acetic acid (15 mL), SeO_2 (0.275 g, 2.32 mmol) was added, and the reaction mixture was refluxed for 12 h. After reaction completion, the mixture was diluted with 50 mL of DCM and filtered through the pad of Celite®. Further, the mixture was washed with saturated $NaHCO_3$ solution and water. After drying and evaporation of the solvent in vacuo, the crude product was obtained and purified by column chromatography (petroleum ether/acetone 9:1, R_f: 0.30). Yield: 69% (0.274 g), white crystals from acetone/hexane, mp 154 °C.

IR (KBr) ṽ = 2934, 2878, 1724, 1675 cm^{-1}.

^{1}H NMR (400 MHz, $CDCl_3$): 0.95 (s, 3H, H-18); 1.04 (d, J=6.5 Hz, 3H, H-21); 1.24 (s, 3H, H-19); 1.28 (t, J=7.1 Hz, 3H, -O-CH_2**CH_3**,); 2.03 (s, 6H, 2 × **CH_3**CO-); 4.14 (q, J=7.1 Hz, 2H, -O-**CH_2**CH_3); 4.62 (m, 1H, H-3); 5.19 (d, J=3.3 Hz, 1H, H-7); 5.86 (d, J=2.3 Hz, 1H, H-11).

^{13}C NMR (101 MHz, $CDCl_3$): 10.69; 14.26; 19.34; 21.33; 21.39; 23.61; 27.46; 27.72; 30.10; 30.62; 30.83; 31.67; 34.92; 35.32; 36.16; 39.97; 40.70; 41.02; 46.70; 47.06; 53.28; 60.21; 70.16; 73.72; 125.08 (CH, C-11); 159.73 (Cq, C-9); 170.02 (Cq, CH_3CO-); 170.67 (Cq, CH_3CO-); 174.22 (Cq, C-24); 204.50 (Cq, C-12).

Ethyl 3α-acetoxy-7α-hydroxy-5β-cholan-24-oate (10)

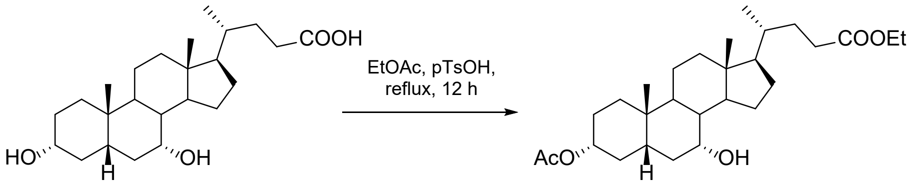

9 **10**

Chenodeoxycholic acid (**9**, 3.00 g, 7.64 mmol) was dissolved in ethyl acetate (75 mL) in a round-bottom flask. *p*-Toluenesulfonic acid (0.306 g, 1.77 mmol) and water (1 mL) were added, and the reaction mixture was refluxed for 12 h. After reaction completion, the mixture was poured into saturated $NaHCO_3$ solution (150 mL). The phases were

separated, and the water phase was extracted with ethyl acetate (3 × 50 mL). The combined organic extracts were washed with water, dried, and the solvent was evaporated in vacuo, giving the crude product, which was purified by column chromatography (toluene/acetone 9:1, R_f: 0.38). Yield: 66% (2.35 g), white amorphous crystals. Spectroscopic data are in good correlation with the literature data [1].

Ethyl 3α-acetoxy-7-oxo-5β-cholan-24-oate (11)

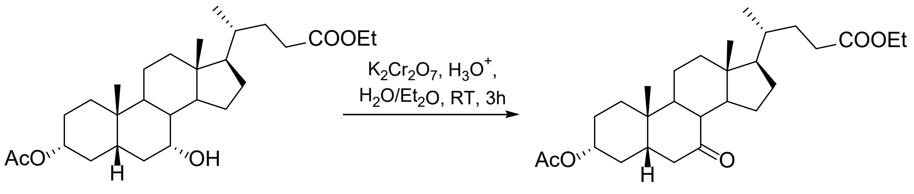

10 **11**

Compound **10** (1.00 g, 2.16 mmol) was dissolved in diethyl ether (25 mL). An acidified solution of $K_2Cr_2O_7$ (0.95 g in 10 mL H_2O) was added dropwise with vigorous stirring. The reaction mixture was kept at room temperature for 3 h with vigorous stirring. After reaction completion, the mixture was transferred to the separation funnel, and the phases were separated. The water phase was extracted with diethyl ether (3 × 20 mL), and the combined organic phases were washed with saturated $NaHCO_3$ solution and water. After drying and evaporation of the solvent in vacuo, the product was obtained and used further without additional purification. Yield: 84% (0.890 g), white amorphous crystals.

R_f: 0.72 (toluene/acetone 4:1) IR (KBr) ṽ = 2958, 2878, 1728 cm^{-1}

^1H NMR (400 MHz, $CDCl_3$): 0.67 (s, 3H, H-18); 0.93 (d, J=6.2 Hz, 3H, H-21); 1.21 (s, 3H, H-19); 1.26 (t, J=7.9 Hz, 3H, -O-CH_2**$\underline{CH_3}$**); 2.00 (s, 3H, **$\underline{CH_3}$**CO-); 4.13 (q, J=7.9 Hz, 2H, -O-**$\underline{CH_2}$**CH_3); 4.69 (m, 1H, H-3).

^{13}C NMR (101 MHz, $CDCl_3$): 12.05; 14.27; 18.38; 21.32; 21.72; 23.04; 24.79; 26.05; 28.28; 30.99; 31.33; 33.11; 33.83; 35.17; 35.21; 38.93; 42.64; 42.76; 45.26; 45.86; 48.89; 49.50; 54.81; 60.21; 72.95 (CH, C-3); 170.57 (Cq, CH_3CO-); 174.25 (Cq, C-24); 211.80 (Cq, C-7).

General procedure for the preparation of hydrazoic acid solution

Benzene solution of hydrazoic acid was prepared in accordance with the literature procedure [2] as follows: In a round-bottom flask, sodium azide (3.00 g, 47 mmol) was

dissolved in water (15 mL), benzene was added (23 mL), and the mixture was cooled with an ice water bath. Further, concentrated sulfuric acid (3 mL) was added with vigorous stirring and cooling. After addition was completed, the mixture was stirred for additional 15 minutes with cooling. The cold mixture was transferred to the separation funnel, and the phases were separated. The cold organic phase was dried, and the obtained solution was used in the reaction immediately.

Analytical data for protected tetrazoles

Ethyl 3α-acetoxy-12a-aza-12a-homo-tetrazolo[5',1':12,12a]-5β-cholan-24-oate (13)

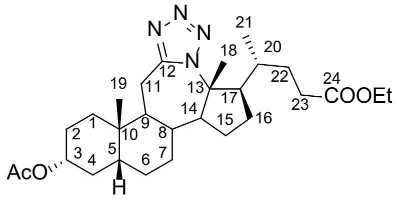

Colorless crystals from ethanol m. p. 194 °C; Rf: 0.55 (toluene/acetone 4:1) IR (KBr) ṽ = 2953, 2872, 1733 cm^{-1}. HRMS (ESI+) *m/z* [M + Na]$^+$ calcd for $[C_{28}H_{44}N_4O_4Na]^+$:523.3255, found: 523.3254. For ^{1}H and ^{13}C NMR data see Table S1.

Ethyl 3α-acetoxy-12a-aza-12a-homo-tetrazolo[5',1':12,12a]-5β-chol-9(11)-en-24-oate (14)

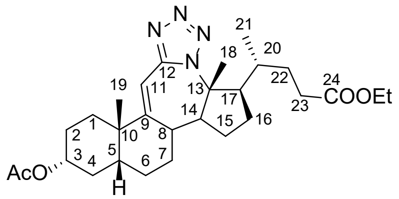

Colorless crystals from ethanol m. p. 197 °C; Rf: 0.51 (toluene/acetone 4:1) IR (KBr) ṽ = 2957, 2872, 1732, 1622 cm^{-1}. HRMS (ESI+) *m/z* [M + Na]$^+$ calcd for $[C_{28}H_{42}N_4O_4Na]^+$: 521.3098 found: 521.3100. For ^{1}H and ^{13}C NMR data see Table S1.

Table S1: Complete assignments of ^1H and ^{13}C chemical shifts for compounds **13** and **14.** Coupling constants are given in Hertz in parentheses.[a]

	compound **13** ($CDCl_3$)		compound **14** ($CDCl_3$)	
position	^1H chemical shift (ppm)	^{13}C chemical shift (ppm)	^1H chemical shift (ppm)	^{13}C chemical shift (ppm)
1	2.10/1.19	35.22 (CH_2)	2.23/1.46	36.06 (CH_2)
2	1.79/1.39[b]	26.54 (CH_2)[b]	1.60/1.43	33.89 (CH_2)
3	4.75, m	73.50 (CH)	4.78, m	73.56 (CH)
4	1.61	31.82 (CH_2)	2.12/1.45	27.59 (CH_2)
5	1.55	41.25 (CH)	1.76	42.35 (CH)
6	1.79/1.64[b]	26.54 (CH_2)[b]	1.92/1.80	27.83 (CH_2)
7	1.36/1.18[b]	26.54 (CH_2)[b]	2.20/1.36	29.23 (CH_2)
8	1.95	39.36 (CH)	2.51	43.19 (CH)
9	1.75	38.61 (CH)	—	156.05 (Cq)
10	—	35.86 (Cq)	—	42.17 (Cq)
11	3.50, H-11α, d (15.1)/2.64, H-11β, dd (15.1, 12.0)	23.52 (CH_2)	6.71, s	108.33 (CH)
12	—	155.46 (Cq)	—	150.63 (Cq)
13	—	69.72 (Cq)	—	69.44 (Cq)
14	1.72	55.96 (CH)	1.97	53.00 (CH)
15	1.96/1.65	21.08 (CH_2)	1.96/1.74	21.32 (CH_2)
16	1.98	26.98 (CH_2)	1.94/1.83	28.14 (CH_2)
17	2.85, td (10.5, 3.2)	52.21 (CH)	2.73, t (10.6)	53.31 (CH)
18	1.47, s	14.66 (CH_3)	1.26, s	14.65 (CH_3)
19	1.06, s	22.94 (CH_3)	1.26, s	29.71 (CH_3)
20	2.19	32.61 (CH)	2.41	31.91 (CH)

21	1.14, d (6.8)	20.18 (CH_3)	1.15, d (6.9)	20.52 (CH_3)
22	1.90/1.42	29.03 (CH_2)	1.36	28.58 (CH_2)
23	2.44/2.32	32.31(CH_2)	2.47/2.32	32.51 (CH_2)
24	—	173.96 (Cq)	—	173.91 (Cq)
**CH_3**CO-	2.0, s	21.36 (CH_3)	2.01, s	21.36 (CH_3)
CH_3**C**O-	—	170.62 (Cq)	—	170.69 (Cq)
-O-CH_2**CH_3**	1.25, t (7.1)	14.23 (CH_3)	1.24, t (7.1)	14.24 (CH_3)
-O-**CH_2**CH_3	4.12, q (7.1)	60.36 (CH_2)	4.11, q (7.1)	60.38 (CH_2)

[a]Multiplicities are determined just for nonoverlapped signals in ^{1}H NMR spectrum. [b]Due to extensive overlap of ^{13}C signals, these assignments could interchange.

Ethyl 12a-aza-3α,7α-diacetoxy-12a-homo-tetrazolo[5',1':12,12a]-5β-cholan-24-oate (15)

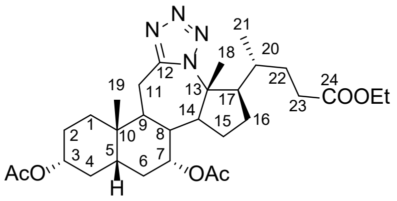

Colorless crystals from ethanol m. p. 207 °C; Rf: 0.45 (toluene/acetone 4:1) IR (KBr) ṽ = 2959, 2874, 1733 cm^{-1}. HRMS (ESI+) *m/z* [M + H]$^+$ calcd for $[C_{30}H_{45}N_4O_6]^+$: 559.3490 found: 559.3497.

^{1}H NMR (400 MHz, $CDCl_3$): 1.07 (s, 3H, H-19); 1.13 (d, J=6.8 Hz, 3H, H-21); 1.24 (t, J = 7.1 Hz, 3H, -O-CH_2**CH_3**); 1.49 (s, 3H, H-18); 2.00 (s, 6H, 2 × **CH_3**CO-); 2.31 (m, 1H, H-23a); 2.45 (m, 1H, H-23b); 2.71 (m, 1H, H-11β); 2.89 (td, J_1 = 10.6 Hz, J_2 = 3.7 Hz, 1H, H-17); 3.59 (d, J = 15.2 Hz, 1H, H-11α); 4.11 (q, J = 7.1 Hz, 2H, -O-**CH_2**CH_3); 4.61 (m 1H, H-3); 4.98 (s, 1H, H-7).

^{13}C NMR (101 MHz, $CDCl_3$): 14.06 (CH_3, C-18); 14.23; 20.02; 21.30; 21.37; 21.50; 22.44 (CH_3, C-19); 22.48 (CH_2, C-11); 26.13; 26.57; 29.04; 30.86; 32.13; 32.70; 33.61; 34.45; 35.42; 36.34; 40.26; 41.40; 50.93; 51.92; 60.38; 69.08; 71.85 (CH, C-7); 73.29 (CH, C-3); 154.92 (Cq, C-12); 170.05 (Cq, CH_3CO-); 170.65 (Cq, CH_3CO-); 173.88 (Cq, C-24).

Ethyl 12a-aza-3α,7α-diacetoxy-12a-homo-tetrazolo[5',1':12,12a]-5β-chol-9(11)-en-24-oate (16)

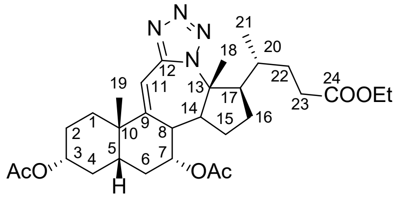

Colorless crystals from ethanol m. p. 201-202 °C; Rf: 0.48 (toluene/acetone 4:1) IR (KBr) ṽ = 2934, 2872, 1734, 1656 cm^{-1}. HRMS (ESI+) *m/z* $[M + Na]^+$ calcd for $[C_{30}H_{44}N_4O_6Na]^+$: 579.3153 found: 579.3157.

^{1}H NMR (400 MHz, $CDCl_3$): 1.14 (d, J=6.8 Hz, 3H, H-21); 1.22 (t, J = 7.2 Hz, 3H, -O-CH_2**CH_3**); 1.29 (s, 6H, H-18, H-19); 1.95 (s, 3H, **CH_3**CO-); 2.01 (s, 3H, **CH_3**CO-); 4.09 (q, J = 7.2 Hz, 2H, -O-**CH_2**CH_3); 4.64 (m 1H, H-3); 5.19 (d, J = 2.7 Hz, 1H, H-7); 6.86 (s, 1H, H-11).

^{13}C NMR (101 MHz, $CDCl_3$): 14.22; 14.29; 20.43; 21.16; 21.30; 21.36; 27.89; 28.22; 28.52; 29.81; 31.62; 31.90; 32.39; 35.88 (2 × CH_2); 41.10; 42.02; 45.52; 49.03; 53.23; 60.36; 69.09; 72.24 (CH, C-7); 73.43 (CH, C-3); 110.88 (CH, C-11); 150.41 (Cq, C-12); 152.03 (Cq, C-9); 170.05 (Cq, CH_3CO-); 170.64 (Cq, CH_3CO-); 173.79 (Cq, C-24).

Ethyl 3α-acetoxy-7a-aza-7a-homo-tetrazolo[5',1':7,7a]-5β-cholan-24-oate (17)

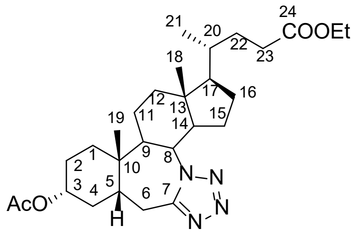

Colorless oil that solidifies on standing, resistant to crystallisation. Rf: 0.40 (toluene/acetone 4:1) IR (KBr) ṽ = 2951, 2878, 1729 cm^{-1}. HRMS (ESI+) *m/z* $[M + H]^+$ calcd for $[C_{28}H_{45}N_4O_4]^+$: 501.3441 found: 501.3443.

^{1}H NMR (400 MHz, $CDCl_3$): 0.45 (q, J = 12.7 Hz, 1H, H-4α); 0.76 (s, 3H, H-18); 0.98 (d, J=6.4 Hz, 3H, H-21); 1.27 (m, 6H, H-19 and -O-CH_2**CH_3**); 1.95 (s, 3H, **CH_3**CO-); 3.09 (dd, J_1 = 15.2 Hz, J_2 = 5.3 Hz, 1H, H-6a); 3.25 (d, J = 15.2 Hz, 1H, H-6b); 4.14 (q, J = 7.2 Hz, 2H, -O-**CH_2**CH_3); 4.25 (t, J = 10.3 Hz, 1H, H-8); 4.72 (m 1H, H-3).

^{13}C NMR (101 MHz, $CDCl_3$): 11.45 (CH_3, C-18); 14.28; 18.37; 21.21; 22.18 (CH_2 and CH_3, C-19); 23.92 (CH_2, C-6); 26.21; 26.46; 27.72; 30.78; 31.22; 31.64 (CH_2, C-4); 35.05; 36.74; 37.62; 37.77; 39.35; 42.63; 42.71; 50.33; 54.80; 59.87 (CH, C-8); 60.28; 72.22 (CH, C-3); 154.46 (Cq, C-7); 170.40 (Cq, CH_3CO-); 174.08 (Cq, C-24).

Methyl 7a-aza-3α,12α-diacetoxy-7a-homo-tetrazolo[5',1':7,7a]-5β-cholan-24-oate (18)

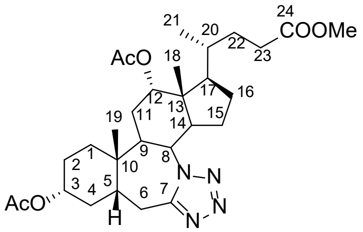

Colorless oil that solidifies on standing, resistant to crystallisation. Rf: 0.46 (toluene/acetone 2:1) IR (KBr) ṽ = 2958, 2878, 1734 cm^{-1}. HRMS (ESI+) *m/z* $[M + Na]^+$ calcd for $[C_{29}H_{44}N_4O_6Na]^+$: 567.3159 found 567.3149.

^1H NMR (400 MHz, $CDCl_3$): 0.48 (q, J = 12.6 Hz, 1H, H-4α); 0.86 (s, 3H, H-18); 0.90 (d, J=6.5 Hz, 3H, H-21); 1.27 (s, 3H, H-19); 1.98 (s, 3H, **CH_3**CO-); 2.16 (s, 3H, **CH_3**CO-); 3.14 (m, 2H, H-6a, H-9); 3.25 (dd, J_1 = 15.3 Hz, J_2 = 2.3 Hz, 1H, H-6b); 3.70 (s, 3H, -O-**CH_3**); 4.31 (t, J = 10.0 Hz, 1H, H-8); 4.73 (m 1H, H-3); 5.22 (s, 1H, H-12).

^{13}C NMR (101 MHz, $CDCl_3$): 12.18 (CH_3, C-18); 17.72; 21.18; 21.36; 22.16 (CH_3, C-19); 23.89 (CH_2, C-6); 25.75; 26.20; 26.92; 27.02; 30.62; 30.88; 31.64; 33.91; 34.30; 36.54; 39.07; 42.46 (CH, C-9); 42.62; 45.11; 46.99; 51.57; 59.36; 72.10 (CH, C-3); 73.26 (CH, C-12); 154.44 (Cq, C-7); 170.27 (Cq, CH_3CO-); 170.57 (Cq, CH_3CO-); 174.35 (Cq, C-24).

Analytical data for free bile acid tetrazoles

12a-Aza-3α-hydroxy-12a-homo-tetrazolo[5',1':12,12a]-5β-cholan-24-oic acid (19)

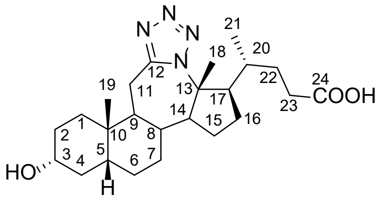

White crystals from methanol m. p. 234 °C; Rf: 0.23 (chloroform/acetone 2:1 + 1% CH_3COOH) IR (KBr) ṽ = 3421, 2935, 2869, 1727 cm^{-1}. HRMS (ESI+) *m/z* $[M + H]^+$ calcd for $[C_{24}H_{39}N_4O_3]^+$: 431.3017 found: 431.3002.

^{1}H NMR (400 MHz, DMSO-d6): 0.98 (s, 3H, H-19); 1.03 (d, J=6.6 Hz, 3H, H-21); 1.42 (s, 3H, H-18); 2.73 (m, 2H, H-17, H-11β); 3.27 (d, 1H, H-11α overlapping with H_2O); 3.41 (m, 1H, H-3, overlapping with H_2O); 4.45 (d, J = 4.2 Hz, 1H, -O**H**); 12.04 (br. s, 1H, -COO**H**).

^{13}C NMR (101 MHz, DMSO-d6): 14.52 (CH_3, C-18); 20.57; 20.90; 23.07 (CH_2, C-11); 23.16 (CH_3, C-19); 26.56; 26.67; 26.90; 29.17; 30.71; 32.29; 32.37; 35.73; 35.90; 36.21; 38.50; 39.07; 41.40; 52.29 (CH, C-17); 55.64; 69.98; 70.05 (CH, C-3); 156.19 (Cq, C-12); 175.19 (Cq, C-24).

12a-Aza-3α,7α-dihydroxy-12a-homo-tetrazolo[5',1':12,12a]-5β-cholan-24-oic acid (20)

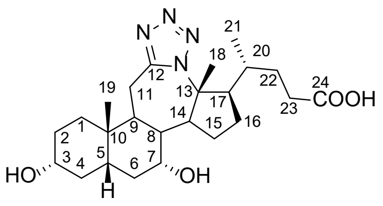

White crystals from methanol m. p. 230-231 °C; Rf: 0.10 (chloroform/acetone 2:1 + 1% CH_3COOH) IR (KBr) ṽ = 3322, 2931, 2869, 1734 cm^{-1}. HRMS (ESI+) *m/z* $[M + Na]^+$ calcd for $[C_{24}H_{38}N_4O_4Na]^+$: 469.2785 found: 469. 2783.

^{1}H NMR (400 MHz, DMSO-d6): 0.98 (s, 3H, H-19); 1.03 (d, J=6.8 Hz, 3H, H-21); 1.44 (s, 3H, H-18); 2.72 (td, J_1 = 10.3 Hz, J_2 = 3.8 Hz, 1H, H-17); 2.81 (m, 1H, H-11β); 3.23 (m, 1H, H-3, overlapping with H_2O); 3.31 (m, 1H, H-11α, overlapping with H_2O); 3.76 (s, 1H, H-7, partial overlapping with H_2O); 4.35 (s, 2H, 2 × -O**H**); 12.01 (br. s, 1H, -COO**H**).

^{13}C NMR (101 MHz, DMSO-d6): 14.00 (CH_3, C-18); 20.36; 21.47; 22.09 (CH_2, C-11); 22.71 (CH_3, C-19); 25.87; 29.35; 30.77; 32.26; 32.67; 32.71; 35.02; 36.13; 36.64; 39.90; 41.19; 42.60; 51.43; 52.02; 67.17 (CH, C-7); 69.41; 70.45 (CH, C-3); 155.98 (Cq, C-12); 175.21 (Cq, C-24).

12a-Aza-3α-hydroxy-12a-homo-tetrazolo[5',1':12,12a]-5β-chol-9(11)-en-24-oic acid (21)

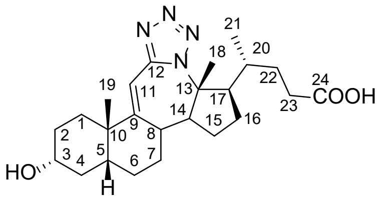

White crystals from methanol m. p. 212-213 °C; Rf: 0.20 (chloroform/acetone 2:1 + 1% CH_3COOH) IR (KBr) ṽ = 3317, 2928, 2869, 1735, 1643 cm^{-1}. HRMS (ESI+) *m/z* $[M + H]^+$ calcd for $[C_{24}H_{37}N_4O_3]^+$: 429. 2860 found: 429. 2844.

^1H NMR (400 MHz, DMSO-d6): 1.07 (d, J=6.7 Hz, 3H, H-21); 1.21 (s, 3H, H-18); 1.23 (s, 3H, H-19); 2.61 (m, 2H, H-5, H-17); 3.48 (m, 1H, H-3, overlapping with H_2O); 4.58 (d, J = 4.5 Hz, 1H, -O**H**); 6.53 (s, 1H, H-11); 12.02 (br. s, 1H, -COO**H**).

^{13}C NMR (101 MHz, DMSO-d6): 14.51 (CH_3, C-18); 20.84; 21.28; 27.78; 28.24; 28.68; 28.72; 29.85 (CH_3, C-19); 31.67; 32.23; 32.50; 36.40; 38.37; 42.32; 42.51; 42.82 (CH, C-5); 52.77; 53.24 (CH, C-17); 69.69; 70.05 (CH, C-3); 107.35 (CH, C-11); 150.86 (Cq, C-12); 157.84 (Cq, C-9); 175.17 (Cq, C-24).

12a-Aza-3α,7α-dihydroxy-12a-homo-tetrazolo[5',1':12,12a]-5β-chol-9(11)-en-24-oic acid (22)

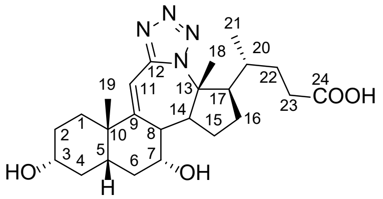

White crystals from methanol m. p. 223 °C; Rf: 0.20 (chloroform/acetone 2:1 + 1% CH_3COOH) IR (KBr) ṽ = 3311, 2928, 2869, 1738, 1643 cm^{-1}. HRMS (ESI+) *m/z* $[M + Na]^+$ calcd for $[C_{24}H_{36}N_4O_4Na]^+$: 467.2629 found: 467.2630.

^1H NMR (400 MHz, DMSO-d6): 1.08 (d, J=6.7 Hz, 3H, H-21); 1.22 (s, 6H, H-18, H-19); 2.66 (m, 2H, H-5, H-17); 3.32 (m, 1H, H-3, overlapping with H_2O); 3.94 (s, 1H, H-7); 4.44 (s, 1H, -O**H**); 4.60 (d, J = 3.0 Hz, 1H, -O**H**); 6.63 (s, 1H, H-11); 12.00 (br. s, 1H, -COO**H**).

^{13}C NMR (101 MHz, DMSO-d6): 14.13 (CH_3, C-18); 20.83; 21.37; 27.58; 28.77; 30.19; 31.86; 32.41; 32.52; 35.35; 36.47; 41.32; 42.31; 42.44; 47.49; 49.59; 53.31; 69.49;

69.54 (CH, C-7); 70.52 (CH, C-3); 109.00 (CH, C-11); 151.01 (Cq, C-12); 156.81(Cq, C-9); 175.20 (Cq, C-24).

7a-Aza-3α-hydroxy-7a-homo-tetrazolo[5',1':7,7a]-5β-cholan-24-oic acid (23)

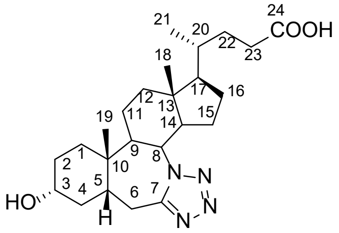

White crystals from ethanol/hexane m. p. 168-169 °C; Rf: 0.17 (toluene/acetone 2:1 + 1% CH_3COOH) IR (KBr) ṽ = 3320, 2931, 2861, 1742 cm^{-1}. HRMS (ESI+) *m/z* $[M + H]^+$ calcd for $[C_{24}H_{39}N_4O_3]^+$: 431.3017 found: 431.3017.

^1H NMR (400 MHz, DMSO-d6): 0.11 (q, J = 11.7 Hz, 1H, H-4α); 0.72 (s, 3H, H-18); 0.94 (d, J=6.5 Hz, 3H, H-21); 1.21 (s, 3H, H-19); 2.85 (dd, J_1 = 14.8 Hz J_2 = 5.7 Hz, 1H, H-6a); 3.38 (m, 1H, H-3, overlapping with H_2O); 3.43 (m, 1H, H-6b overlapping with H_2O); 4.42 (d, J = 4.9 Hz, 1H, -O**H**); 4.58 (t, J = 10.5 Hz, 1H, H-8); 11.98 (br. s, 1H, -COO**H**).

^{13}C NMR (101 MHz, DMSO-d6): 11.63 (CH_3, C-18); 18.66; 22.09; 22.30 (CH_3, C-19); 23.85 (CH_2, C-6); 26.05; 27.80; 30.38; 31.02; 31.13; 35.07; 36.34 (CH_2, C-4); 37.07; 37.47; 37.92; 39.54 (Cq, overlapping with DMSO-d6); 42.32; 42.74; 50.65; 54.76; 59.25 (CH, C-8); 69.30 (CH, C-3); 155.64 (Cq, C-7); 175.35 (Cq, C-24).

7a-Aza-3α,12α-dihydroxy-7a-homo-tetrazolo[5',1':7,7a]-5β-cholan-24-oic acid (24)

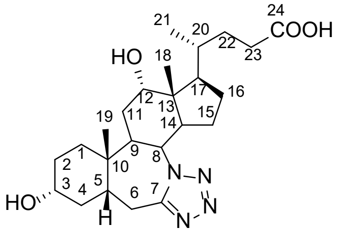

White crystals from ethanol/hexane m. p. 238 °C; Rf: 0.28 (chloroform/acetone 1:1 + 1% CH_3COOH) IR (KBr) ṽ = 3329, 2929, 1739 cm^{-1}. HRMS (ESI+) *m/z* $[M + H]^+$ calcd for $[C_{24}H_{39}N_4O_3]^+$: 447.2971 found: 447.2957.

^1H NMR (400 MHz, DMSO-d6): 0.12 (q, J = 11.9 Hz, 1H, H-4α); 0.69 (s, 3H, H-18); 0.96 (d, J=6.5 Hz, 3H, H-21); 1.18 (s, 3H, H-19); 2.84 2.85 (dd, J_1 = 15.7 Hz J_2 = 5.6 Hz, 1H,

H-6a); 3.00 (m, 1H, H-14); 3.37 (m, 1H, H-3, overlapping with H_2O); 3.41 (m, 1H, H-6b overlapping with H_2O); 3.89 (s, 1H, H-12); 4.47 (br. s, -O**H**); 4.55 (t, J = 10.4 Hz, 1H, H-8); 4.68 (br. s, -O**H**); 11.96 (br. s, 1H, -COO**H**).

^{13}C NMR (101 MHz, DMSO-d6): 12.50 (CH_3, C-18); 17.61; 22.27 (CH_3, C-19); 23.86 (CH_6, C-6); 25.55; 27.31; 30.06; 30.17; 31.11; 31.28; 33.16; 35.05; 36.32 (CH_2, C-4); 37.09; 39.17; 41.15 (CH, C-14); 42.27; 46.06; 46.58; 59.53; 69.33 (CH, C-3); 69.56 (CH, C-12); 155.60 (Cq, C-7); 175.43 (Cq, C-24).

2. X-ray crystallography data

The diffraction data for compounds **13** and **14** were collected on a Rigaku (Oxford Diffraction) Gemini S diffractometer using graphite-monochromated Mo Kα radiation (λ = 0.71071 Å). CrysAlisPro and CrysAlis RED software packages [3] were employed for data collection and data integration. The space group determinations were based on an analysis of the Laue class and the systematically absent reflections. Collected data were corrected for absorption effects using the multiscan method, applying an empirical absorption correction using spherical harmonics [4] as implemented in SCALE3 ABSPACK [3] scaling algorithm.

The structures were solved by direct methods using SHELXT [5]. The structures were refined by full-matrix least-squares procedures on F^2 using SHELXL-2018/3 program [6]. For both compounds, nonhydrogen atoms were refined anisotropically, while the hydrogen atoms attached to carbon atoms were placed in geometrically idealized positions and refined as riding on the parent atoms, with C–H = 0.98 Å and U_{iso}(H) = 1.2·U_{eq} (C) for methine groups, C–H = 0.97 Å and U_{iso} (H) = 1.2·U_{eq} (C) for methylene groups, and with C–H = 0.96 Å and U_{iso} (H) = 1.5·U_{eq} (C) for methyl groups. The crystal data and refinement parameters are summarized in Table S2.

Table S2: Crystallographic data and refinement parameters for compounds **13** and **14**

	**13**	**14**
chemical formula	$C_{28}H_{44}N_4O_4$	$C_{28}H_{42}N_4O_4$
M_r	500.67	498.65
T (K)	293	179
crystal system	orthorhombic	orthorhombic

space group	$P2_12_12_1$	$P2_12_12_1$
a (Å)	7.9904 (6)	7.8084 (8)
b (Å)	15.1903 (13)	15.1919 (13)
c (Å)	23.1138 (19)	23.363 (2)
V (Å^3)	2805.5 (4)	2771.5 (5)
Z	4	4
μ (mm^{-1})	0.08	0.08
θ range (°) for cell measurement	3.7 – 24.5	3.7 – 25.4
crystal size (mm)	0.64 × 0.35 × 0.26	0.57 × 0.31 × 0.24
absorption correction	multiscan	multiscan
T_{min}, T_{max}	0.104, 1.000	0.916, 1.000
reflections collected	6995	12988
independent reflections	4680 (*R* (int) = 0.022)	6455 (*R* (int) = 0.054)
observed ($I > 2\sigma$ (I)) reflections	3771	4145
R ($F^2 > 2\cdot\sigma$ (F^2)), wR (F^2), S	0.071, 0.163, 1.09	0.077, 0.173, 1.02
no. of parameters	330	330
no. of restraints	0	0
$\Delta\rho_{max}$, $\Delta\rho_{min}$ (eÅ^{-3})	0.34, −0.22	0.63, −0.42
CCDC deposition number	2102194	2102193

3. ^{1}H and ^{13}C NMR spectra

Ethyl 3α-acetoxy-12-oxo-5β-holan-24-oate (3)

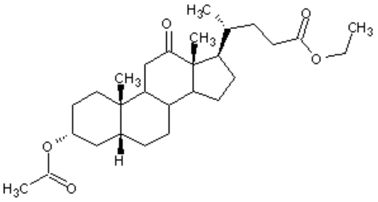

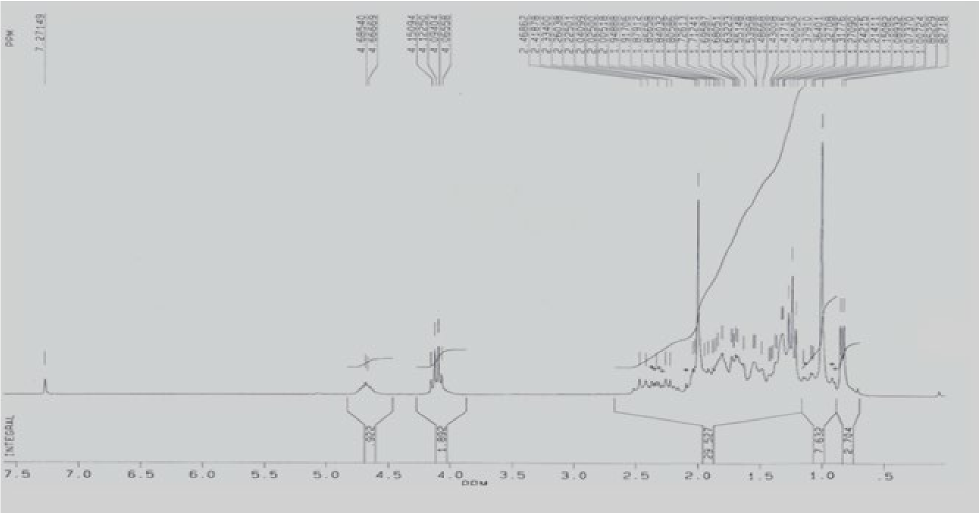

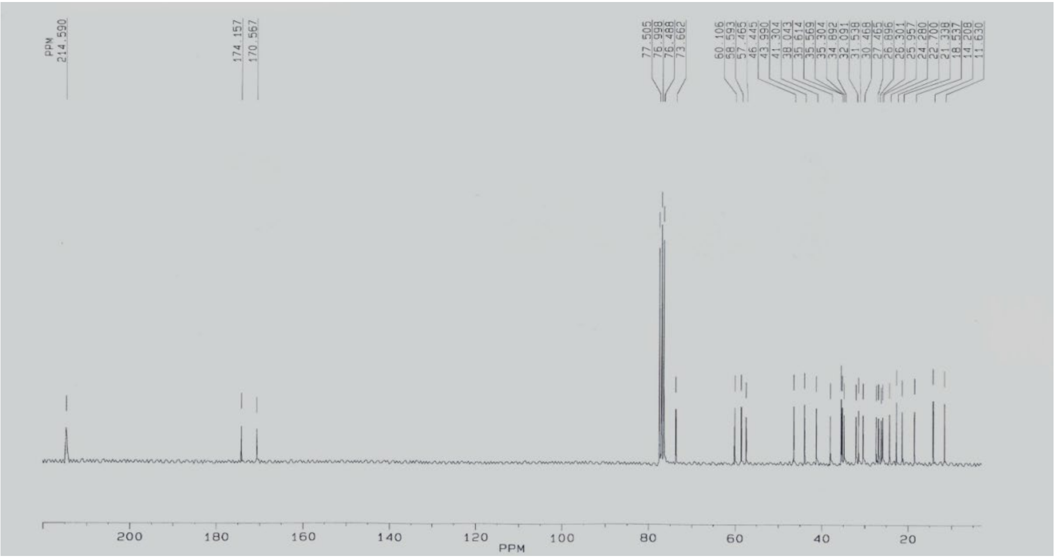

Ethyl 3α-acetoxy-12-oxo-5β-hol-9(11)-en-24-oate (4)

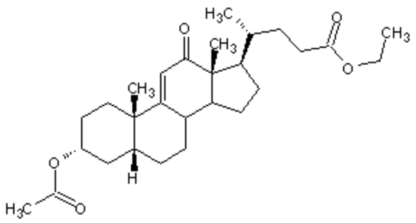

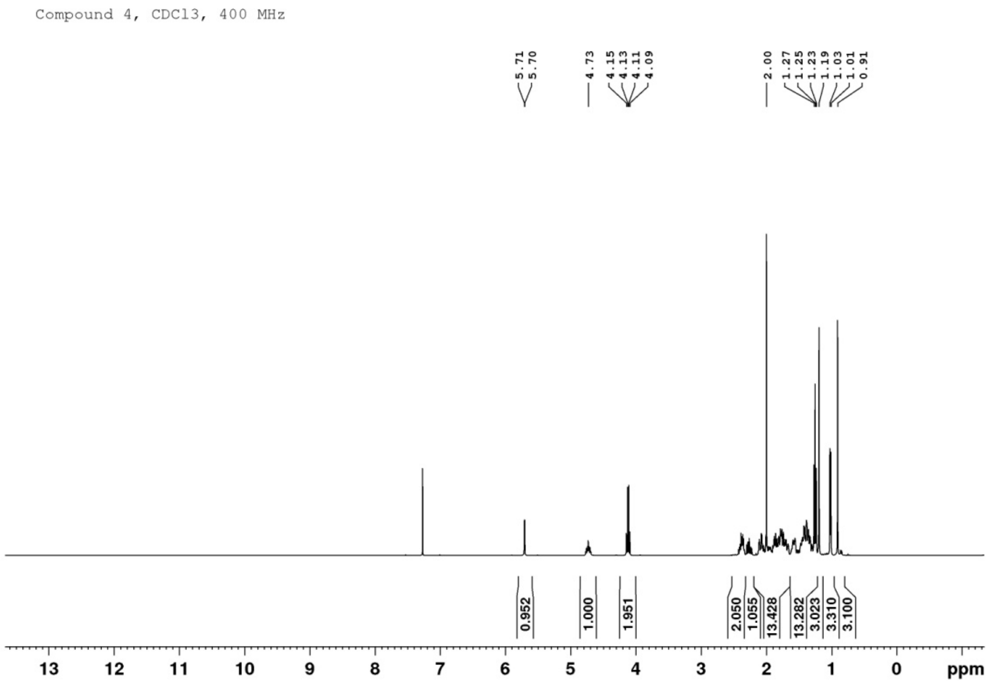

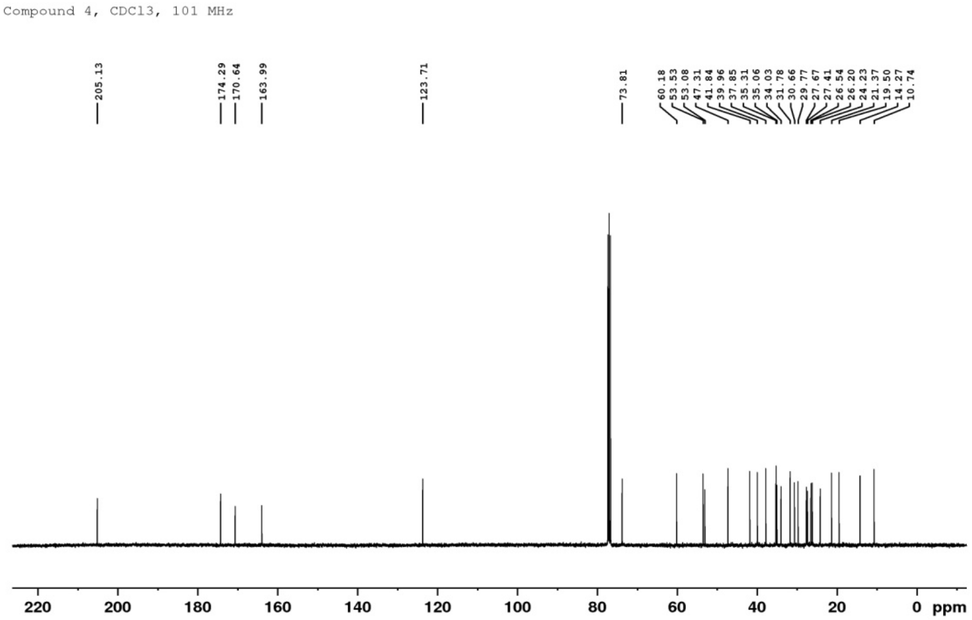

Ethyl 3α,7α-diacetoxy-12-oxo-5β-holan-24-oate (7)

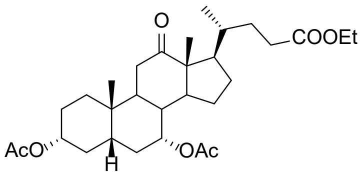

Compound 7, CDCl3, 400 MHz

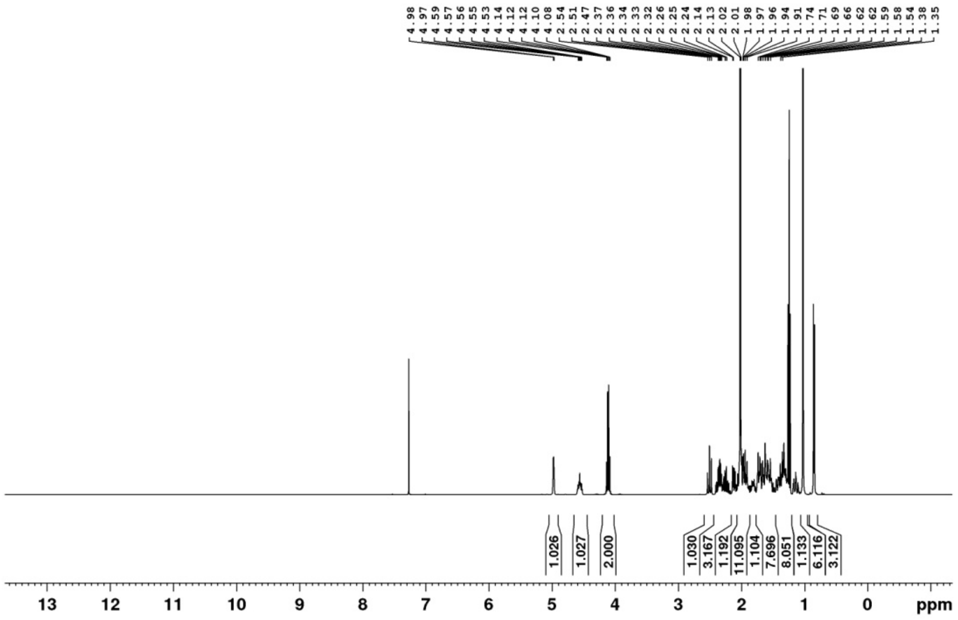

Compound 7, CDCl3, 101 MHz

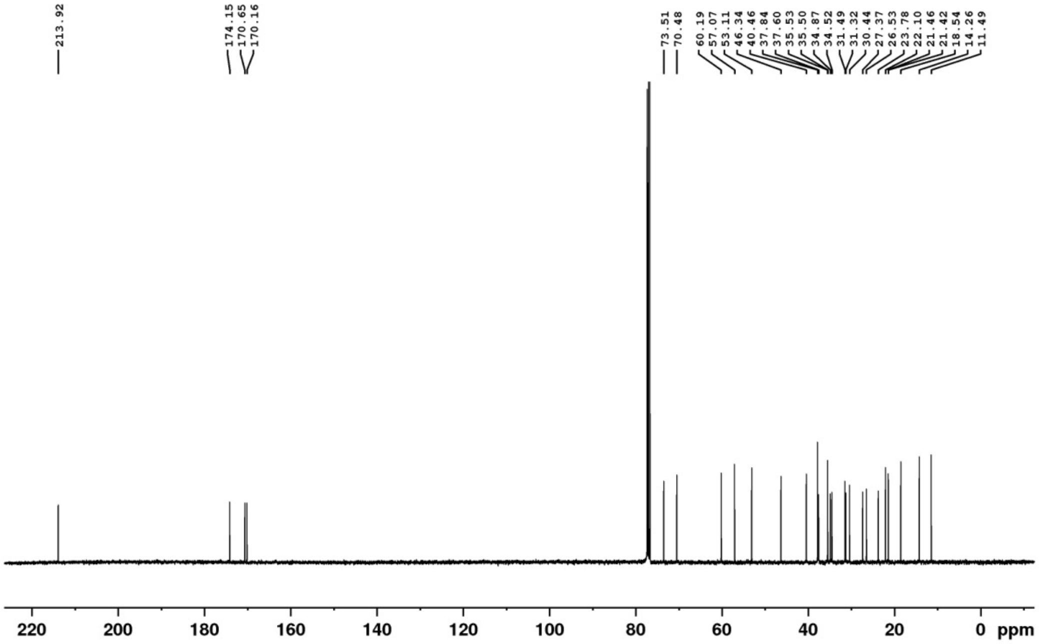

Ethyl 3α,7α-diacetoxy-12-oxo-5β-hol-9(11)-en-24-oate (8)

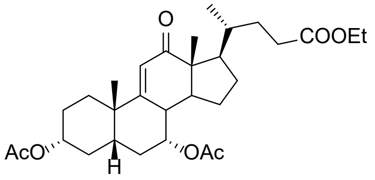

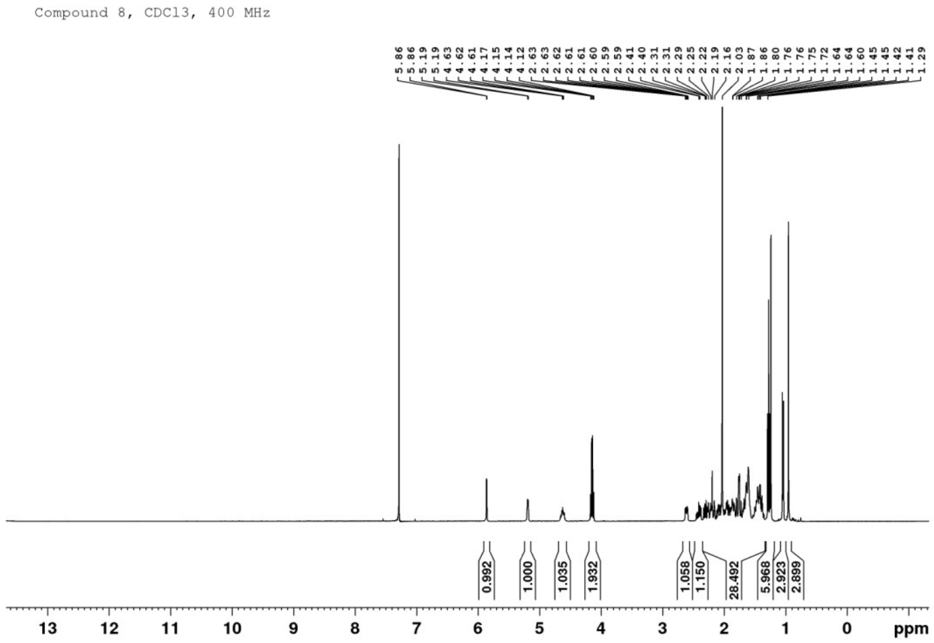

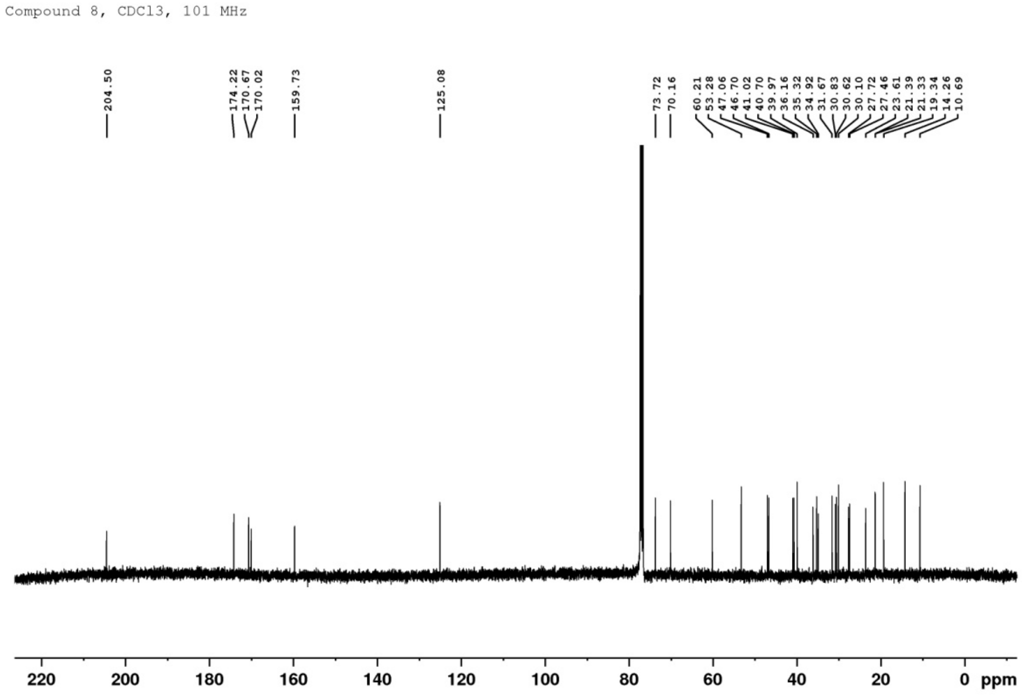

Ethyl 3α-acetoxy-7-oxo-5β-holan-24-oate (11)

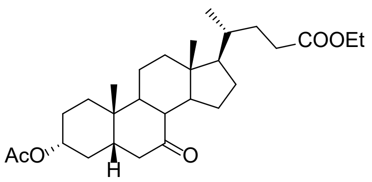

Compound 11, CDCl3, 400 MHz

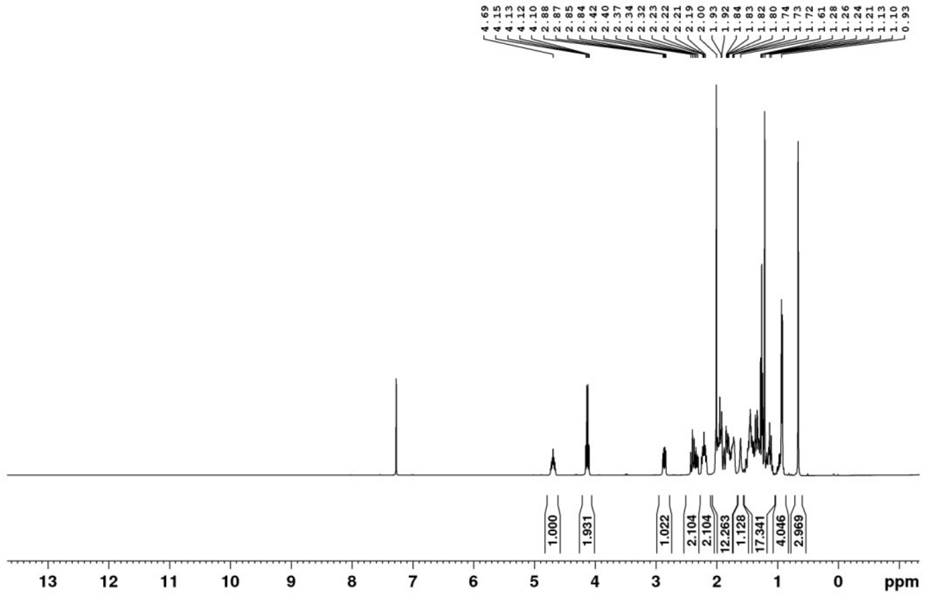

Compound 11, CDCl3, 101 MHz

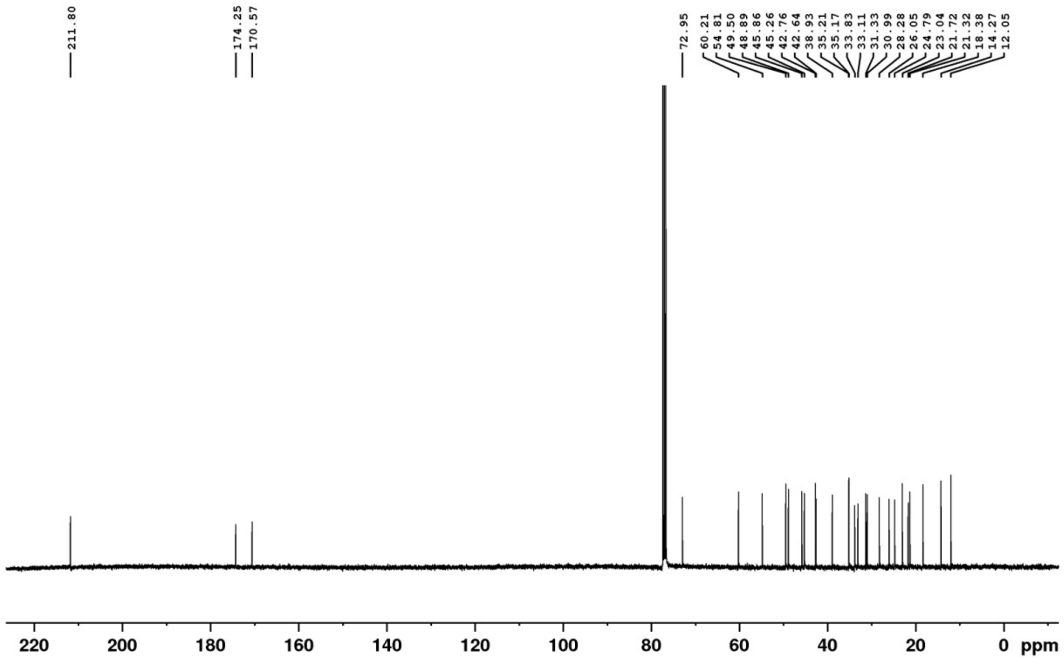

Ethyl 3α-acetoxy-12a-aza-12a-homo-tetrazolo[5’,1’:12,12a]-5β-holan-24-oate (13)

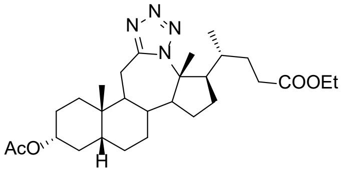

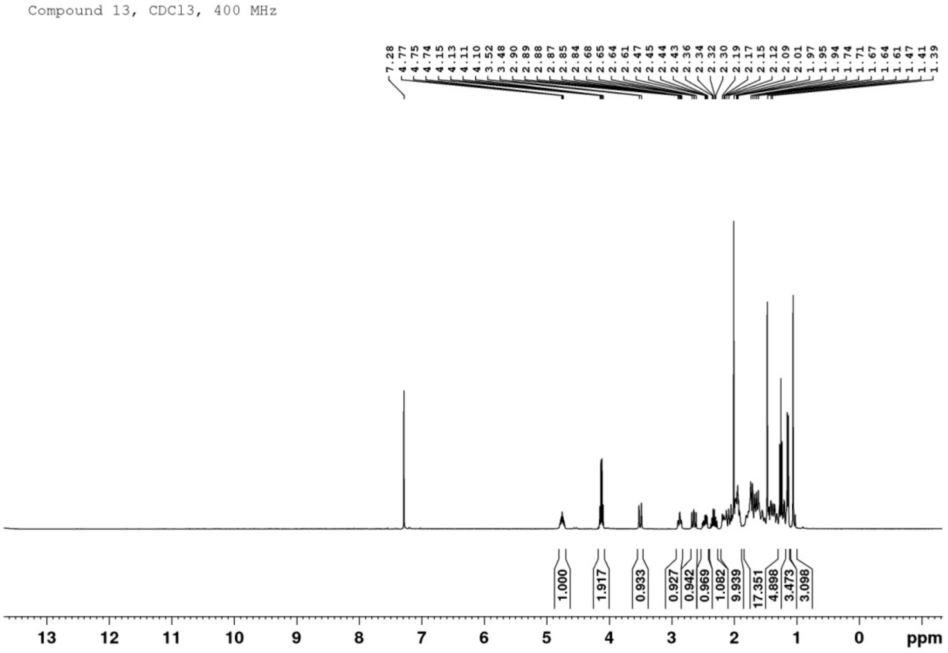

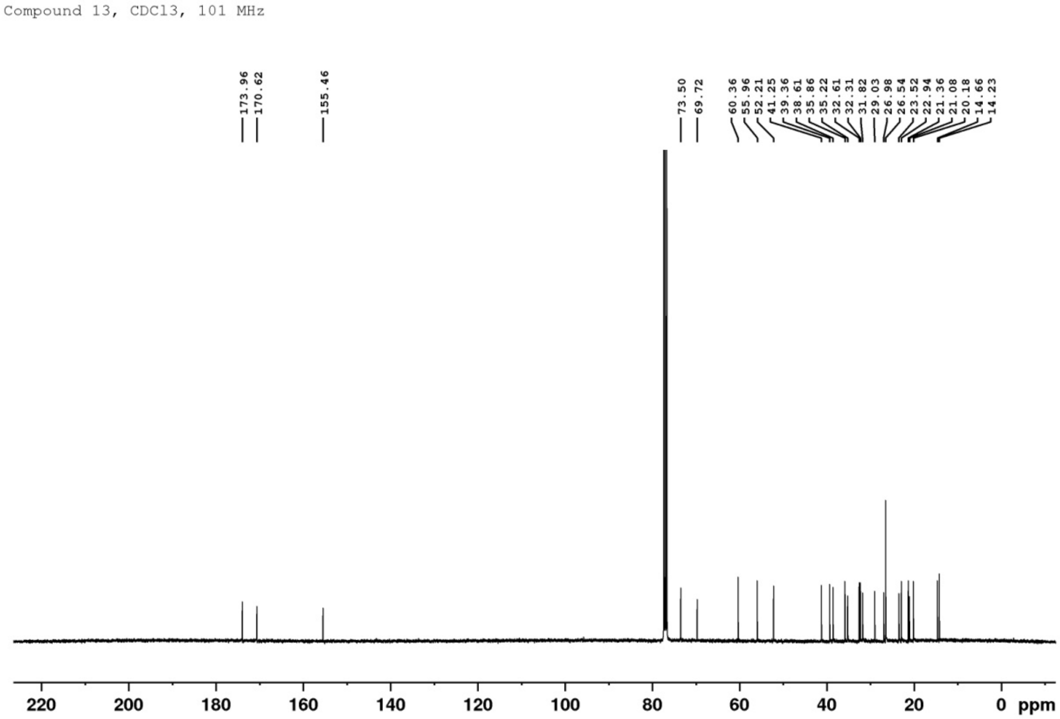

Ethyl 3α-acetoxy-12a-aza-12a-homo-tetrazolo[5',1':12,12a]-5β-hol-9(11)-en-24-oate (14)

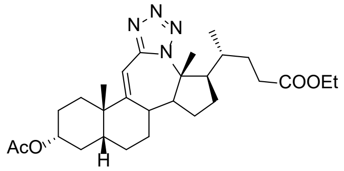

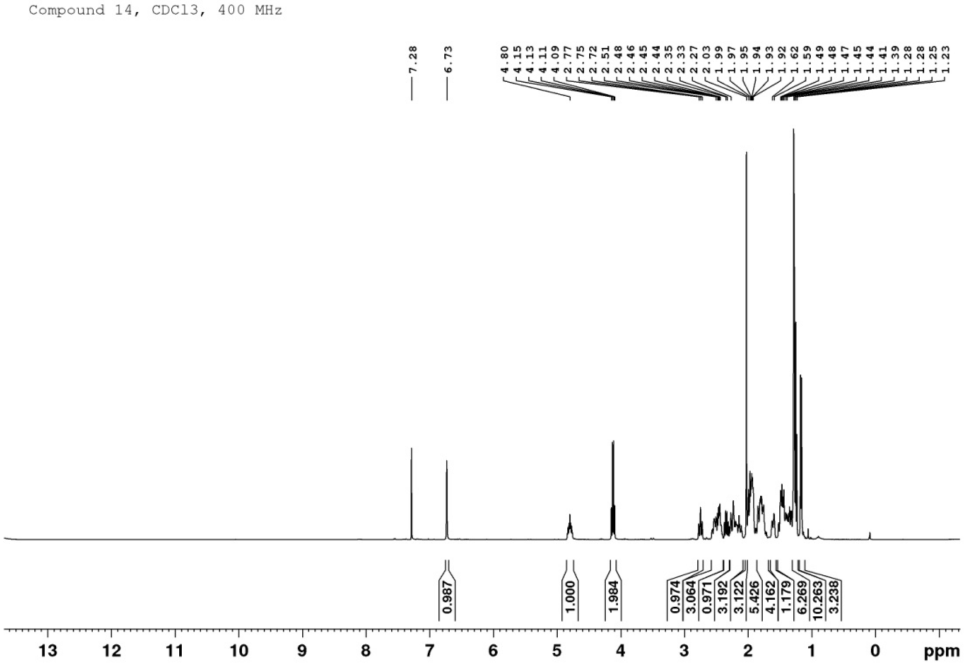

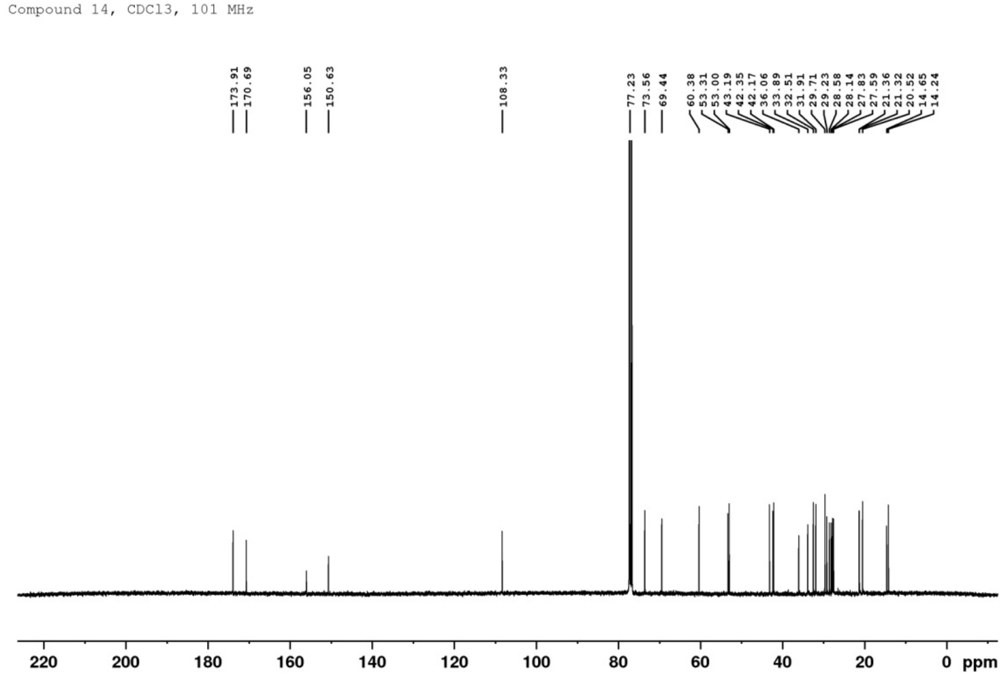

Ethyl 12a-aza-3α,7α-diacetoxy-12a-homo-tetrazolo[5',1':12,12a]-5β-holan-24-oate (15)

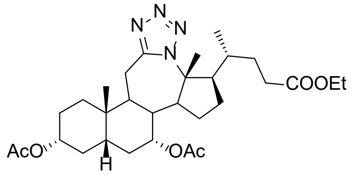

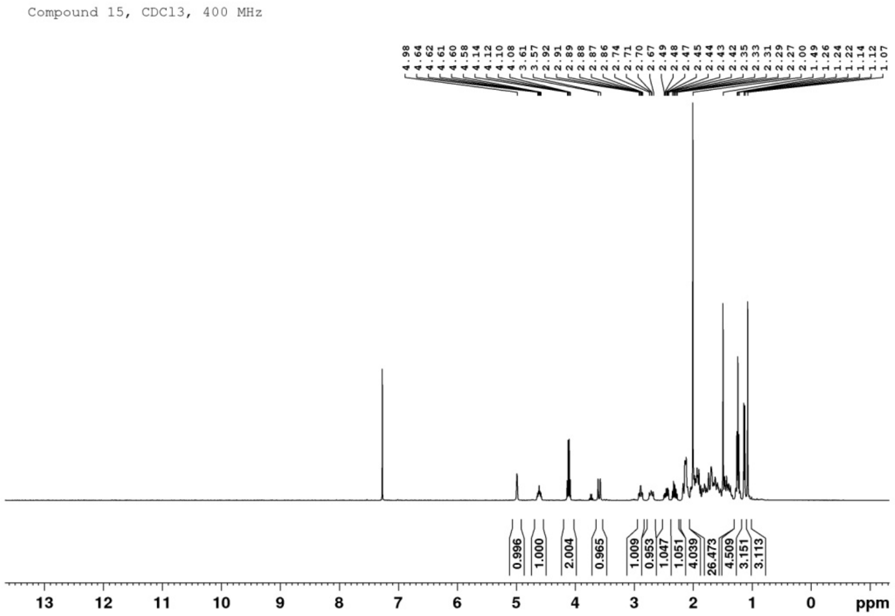

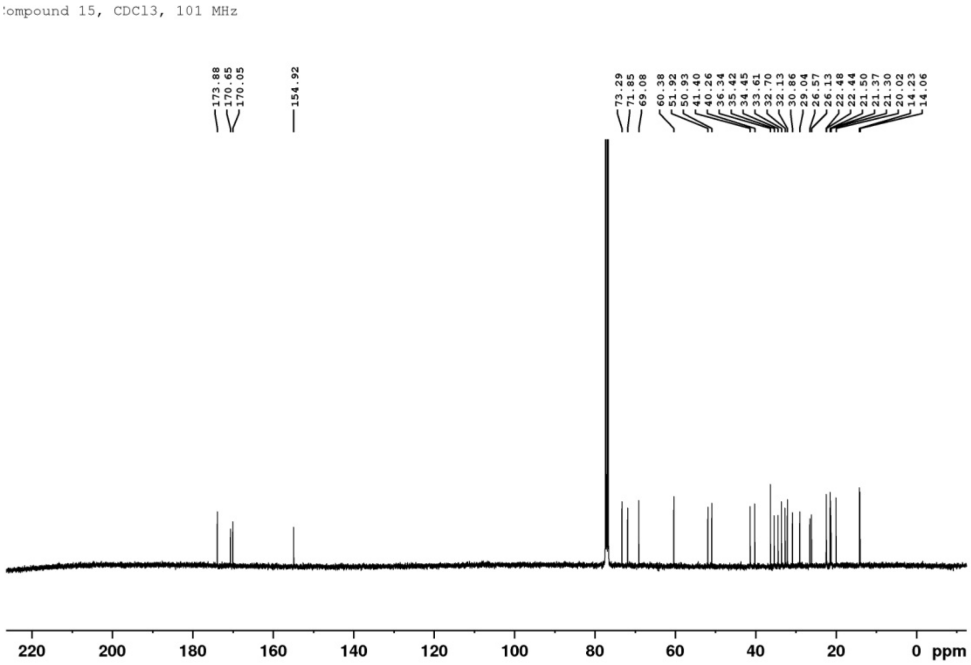

Ethyl 12a-aza-3α,7α-diacetoxy-12a-homo-tetrazolo[5’,1’:12,12a]-5β-hol-9(11)-en-24-oate (16)

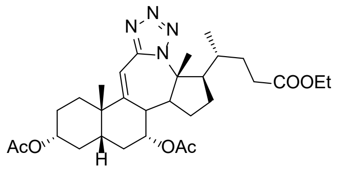

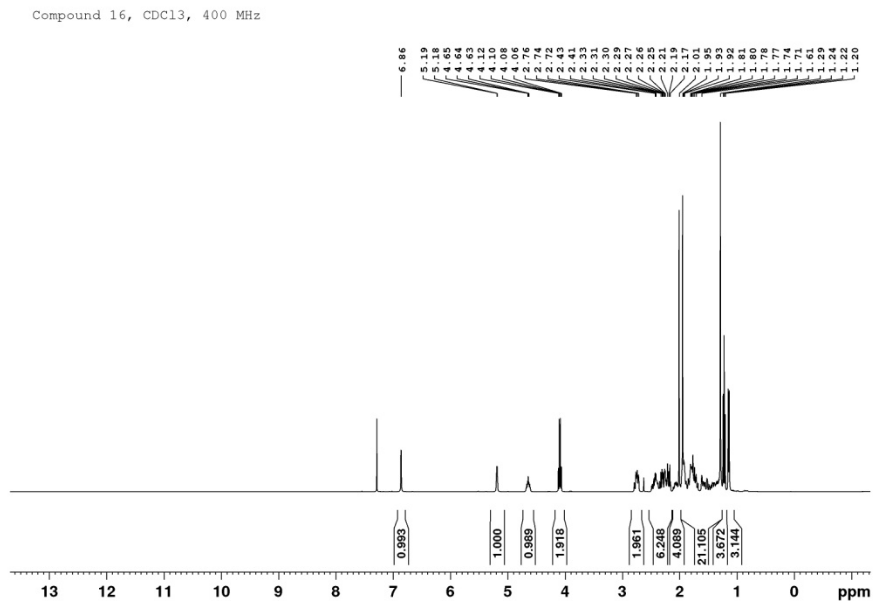

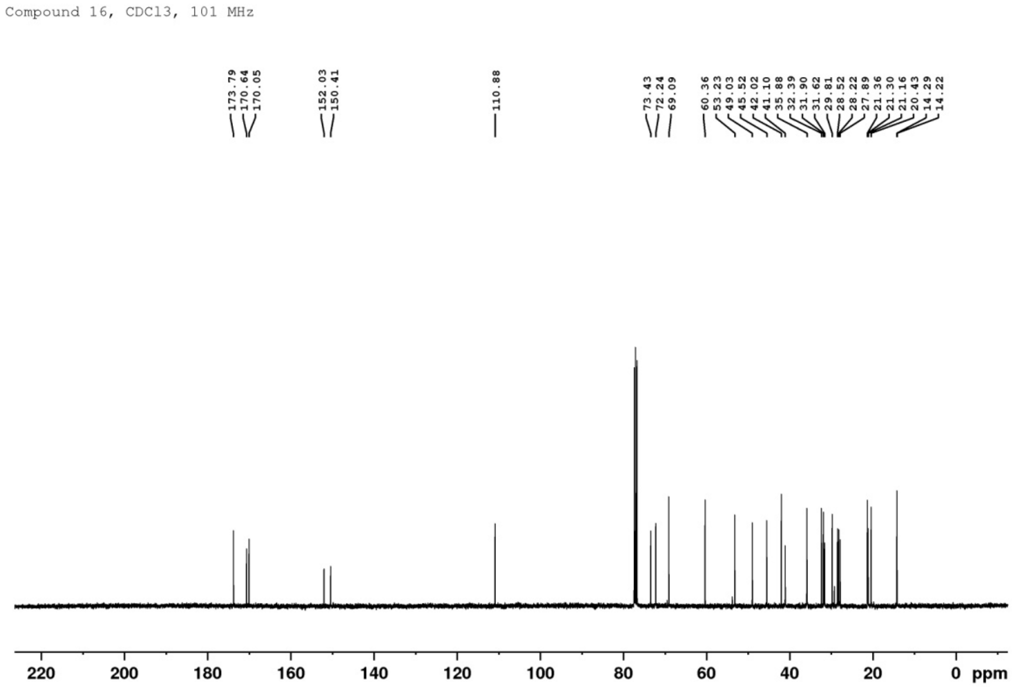

Ethyl 3α-acetoxy-7a-aza-7a-homo-tetrazolo[5',1':7,7a]-5β-holan-24-oate (17)

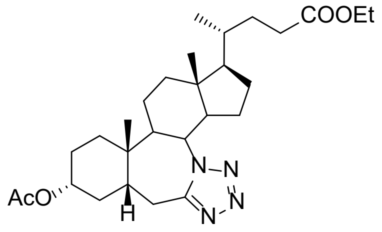

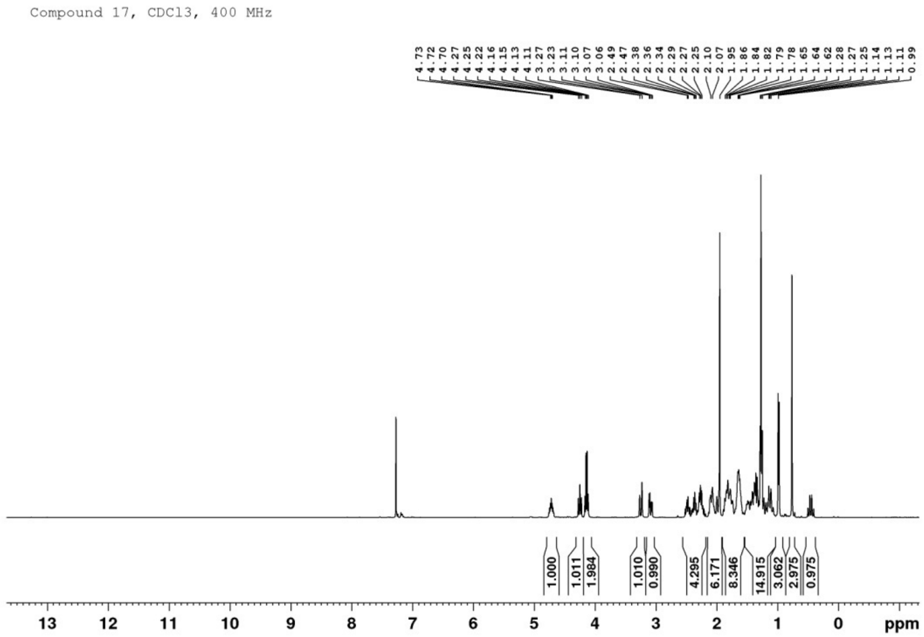

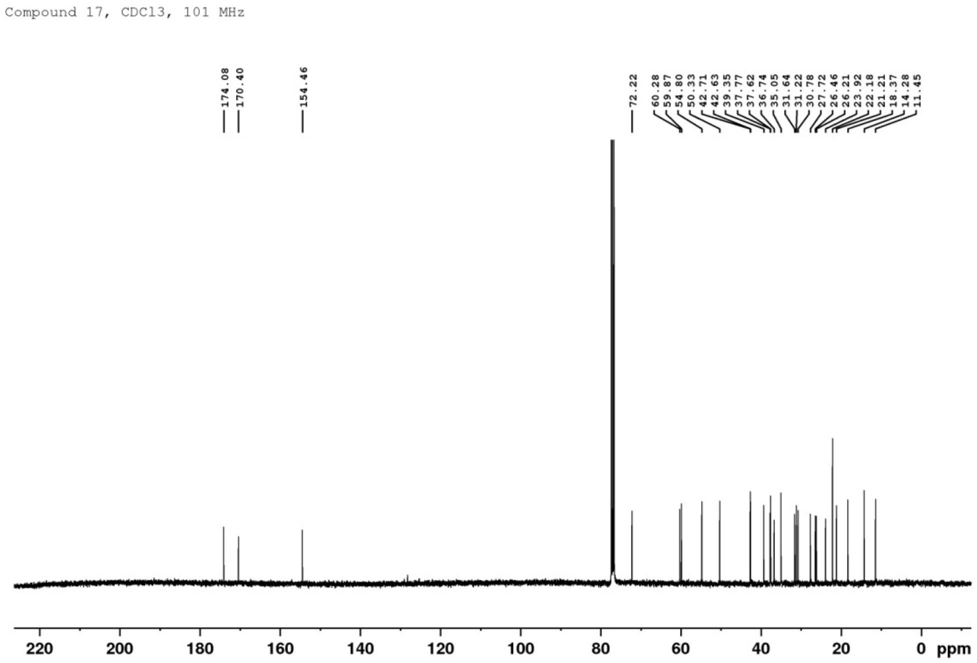

Methyl 7a-aza-3α,12α-diacetoxy-7a-homo-tetrazolo[5',1':7,7a]-5β-holan-24-oate (18)

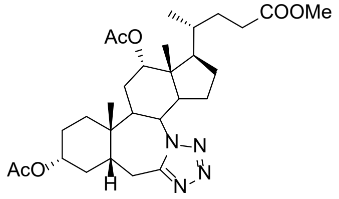

Compound 18, CDCl3, 400 MHz

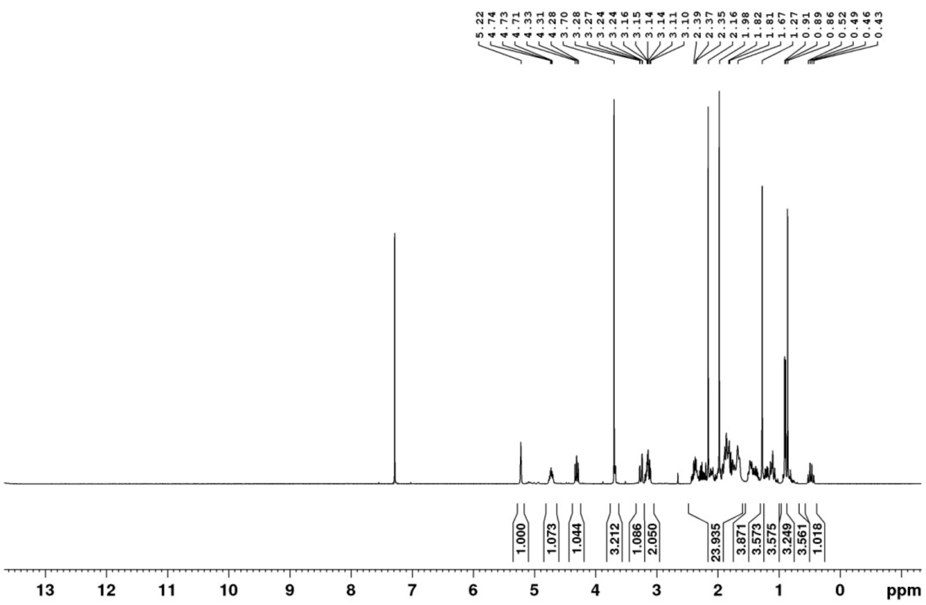

Compound 18, CDCl3, 101 MHz

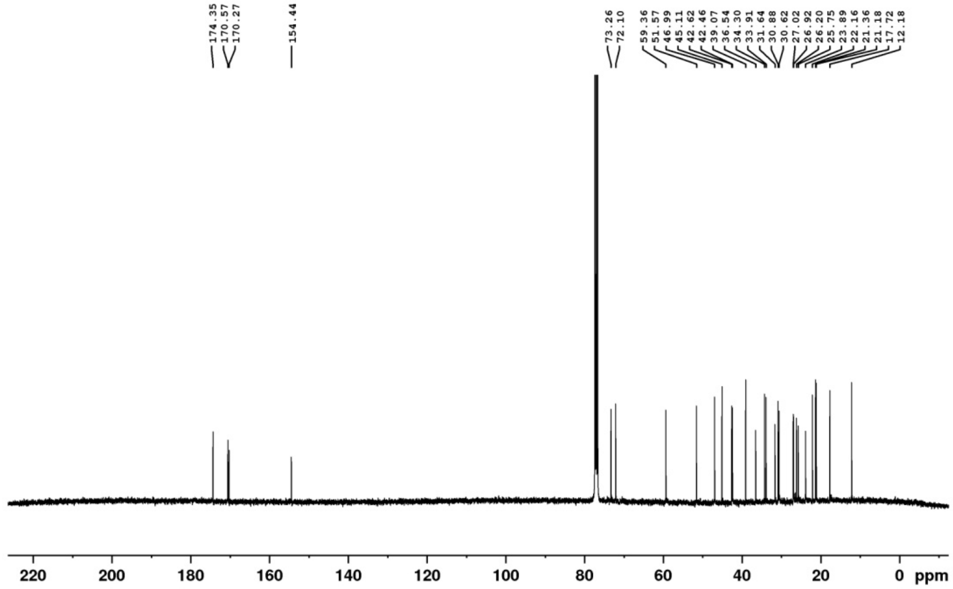

12a-Aza-3α-hydroxy-12a-homo-tetrazolo[5',1':12,12a]-5β-holan-24-oic acid (19)

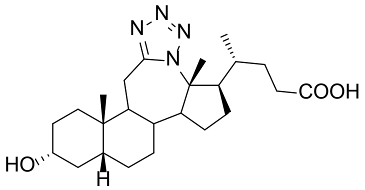

Compound 19, DMSO-d6, 400 MHz

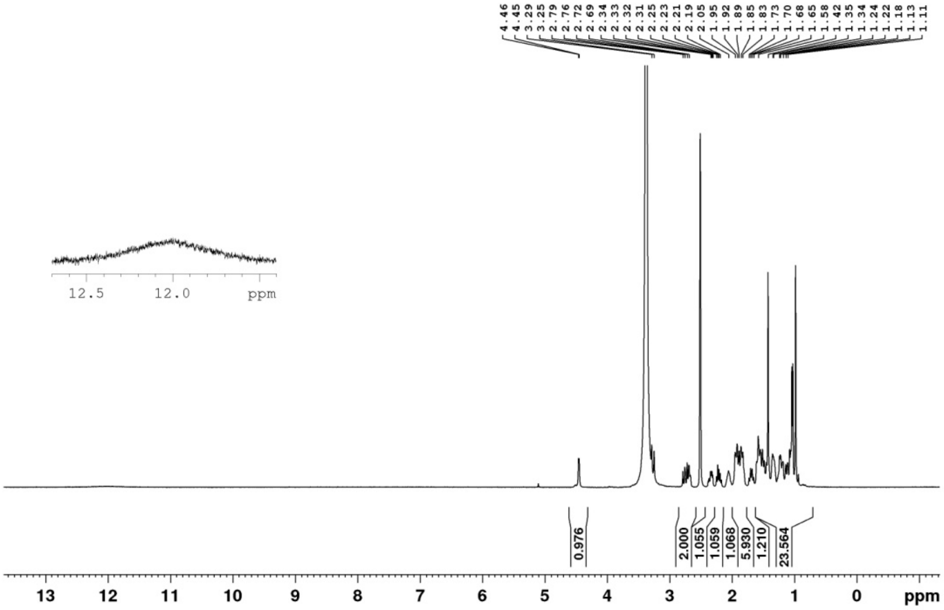

Compound 19, DMSO-d6, 101 MHz

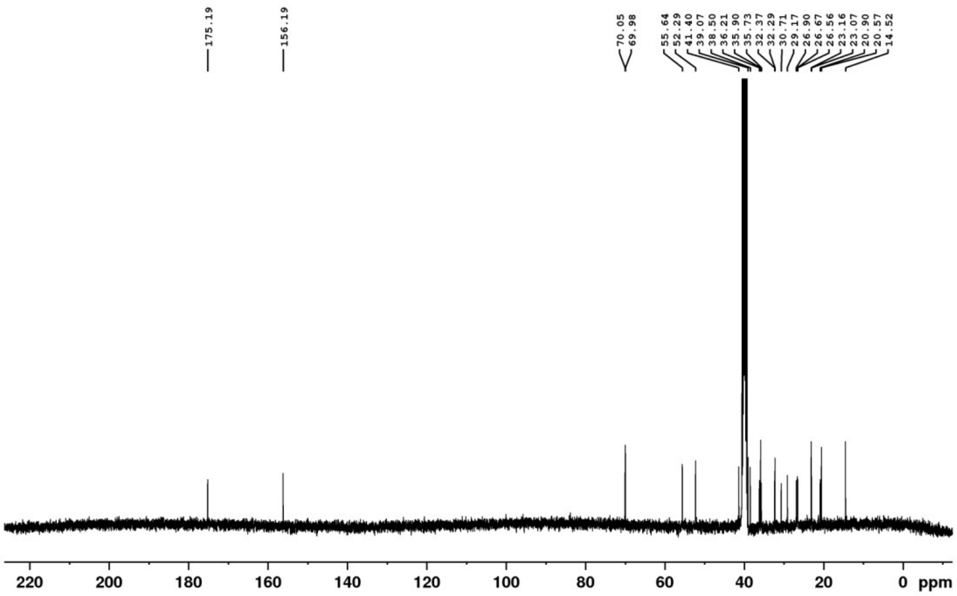

12a-Aza-3α,7α-dihydroxy-12a-homo-tetrazolo[5',1':12,12a]-5β-holan-24-oic acid (20)

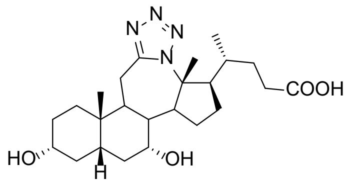

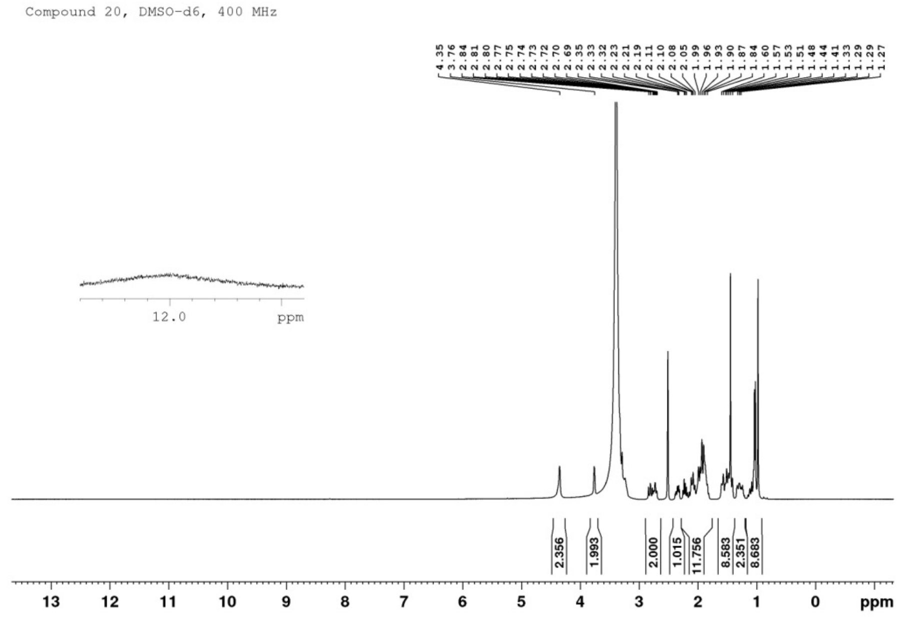

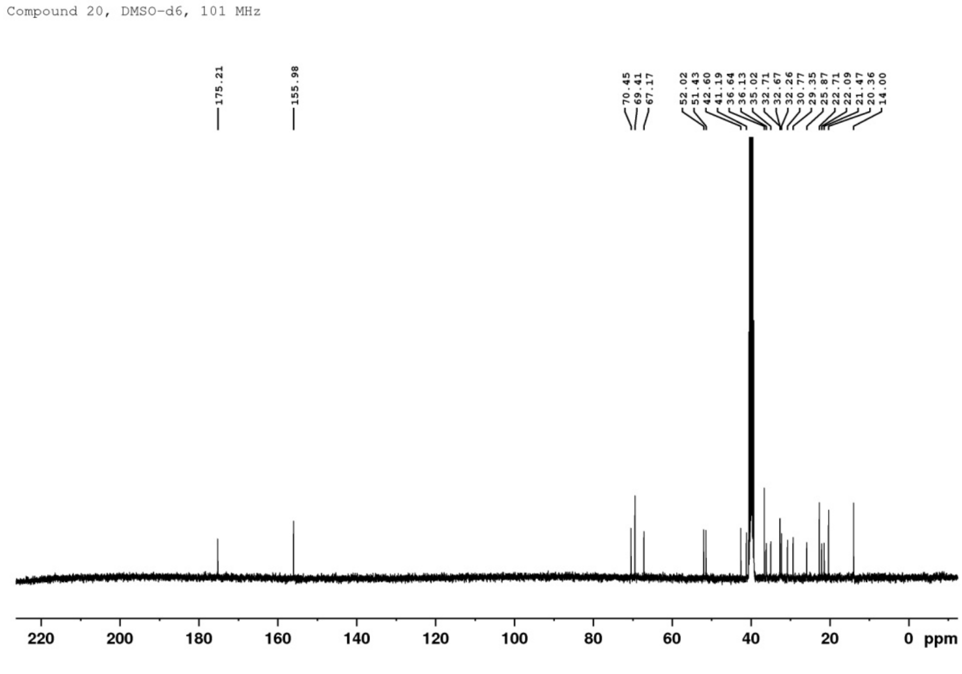

12a-Aza-3α-hydroxy-12a-homo-tetrazolo[5’,1’:12,12a]-5β-hol-9(11)-en-24-oic acid (21)

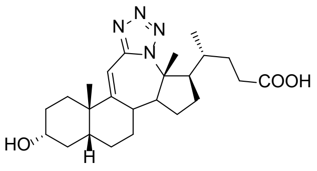

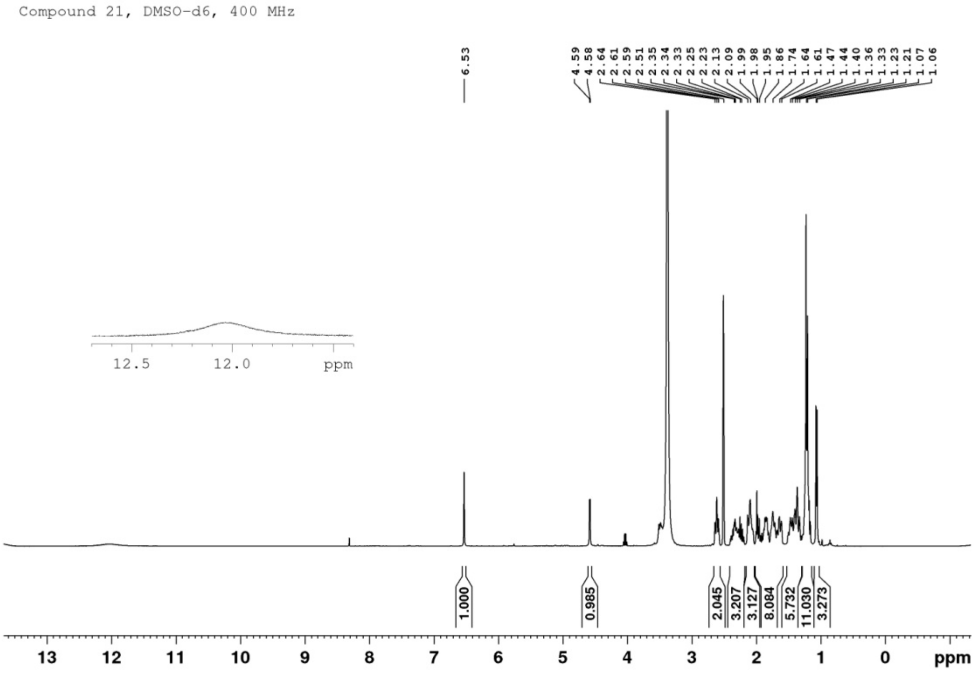

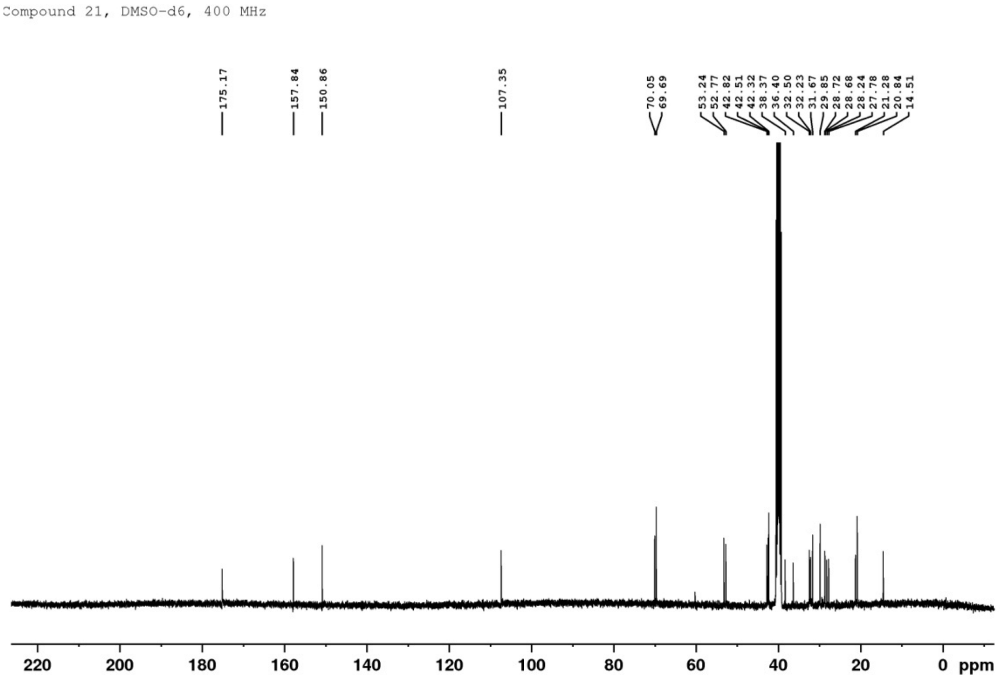

12a-Aza-3α,7α-dihydroxy-12a-homo-tetrazolo[5’,1’:12,12a]-5β-hol-9(11)-en-24-oic acid (22)

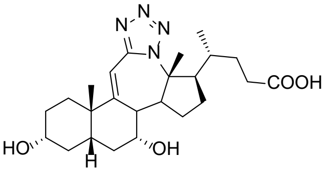

Compound 22, DMSO-d6, 400 MHz

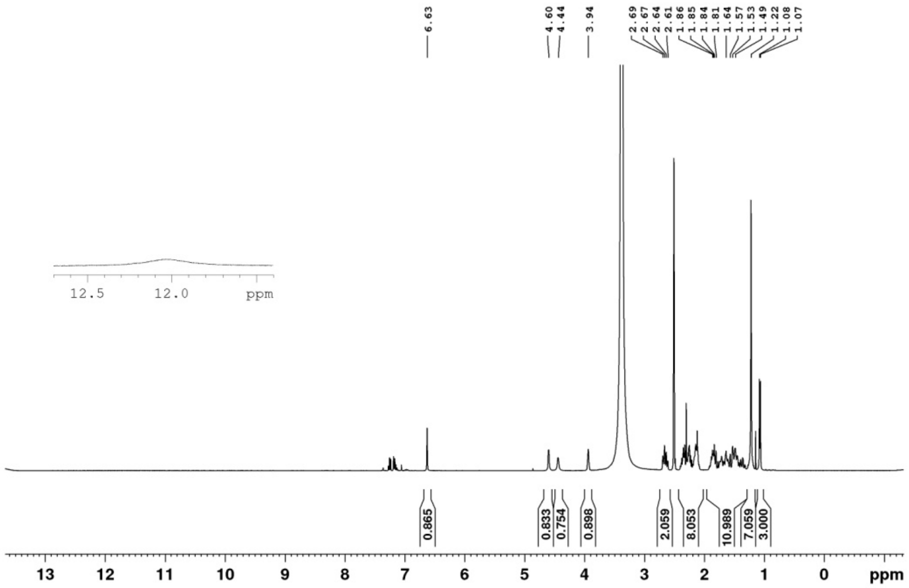

Compound 22, DMSO-d6, 101 MHz

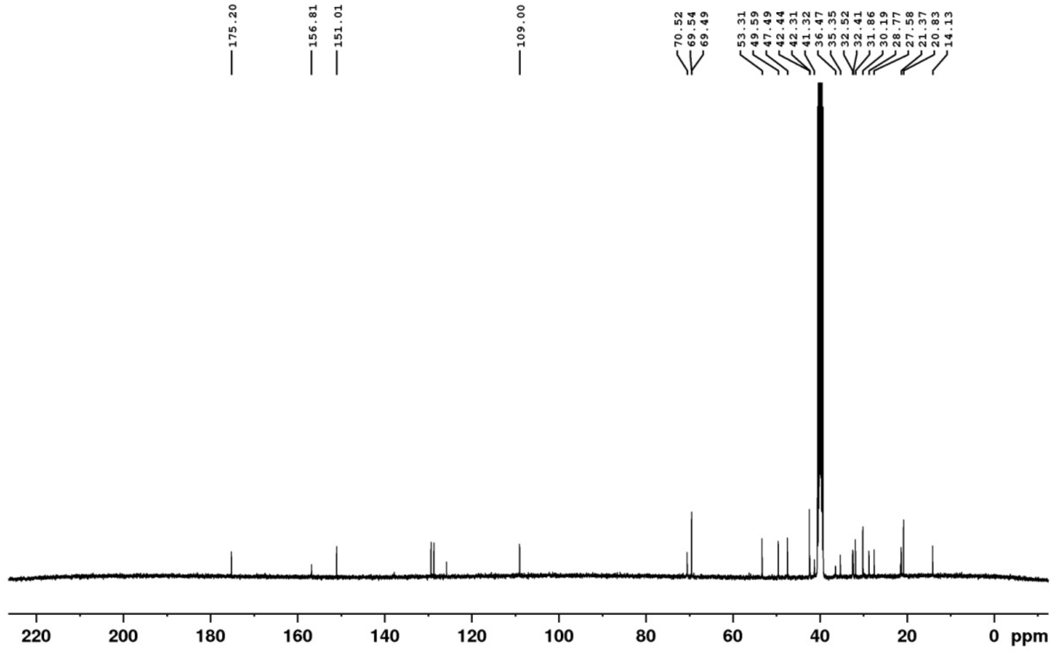

7a-Aza-3α-hydroxy-7a-homo-tetrazolo[5',1':7,7a]-5β-holan-24-oic acid (23)

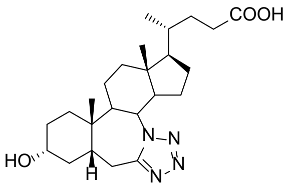

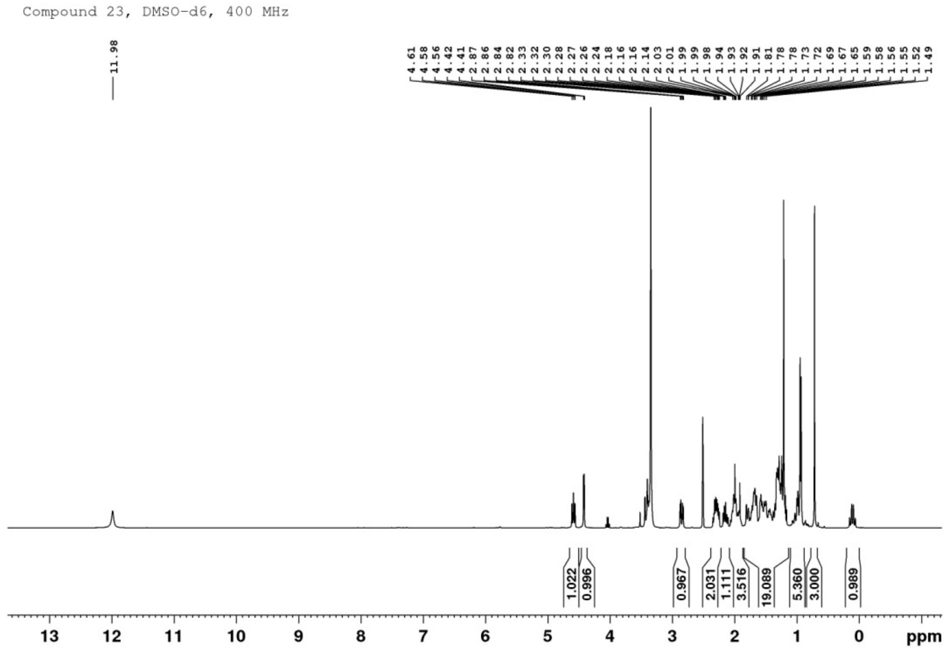

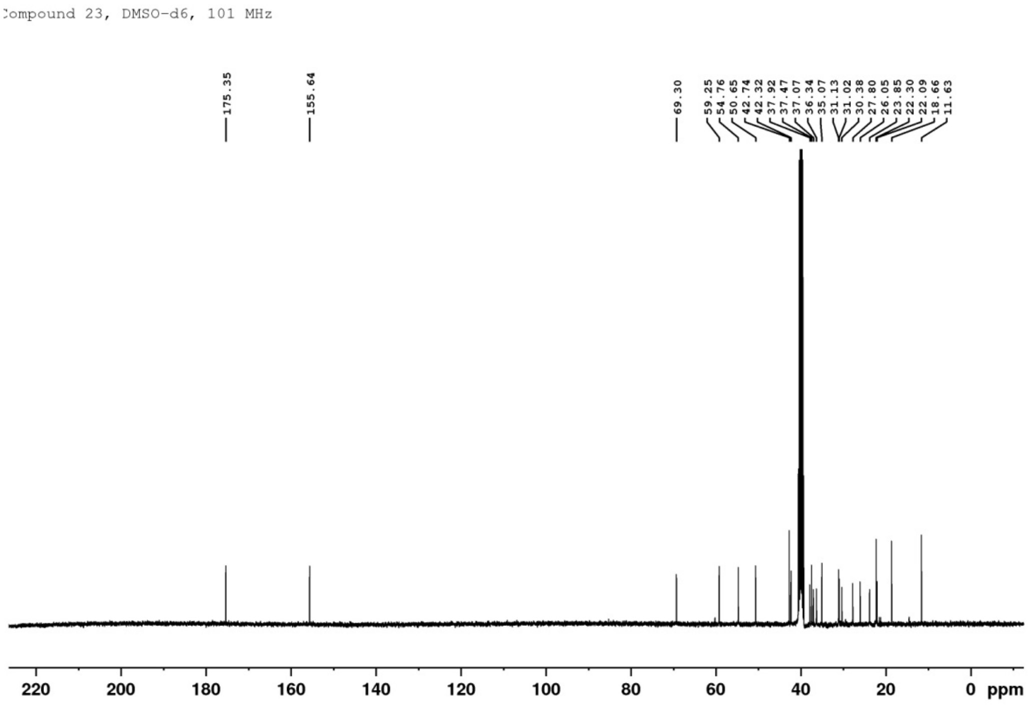

7a-Aza-3α,12α-dihydroxy-7a-homo-tetrazolo[5',1':7,7a]-5β-holan-24-oic acid (24)

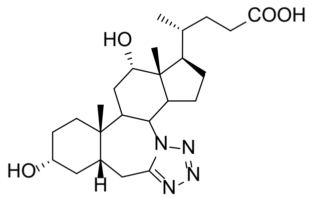

Compound 24, DMSO-D6, 400 MHz

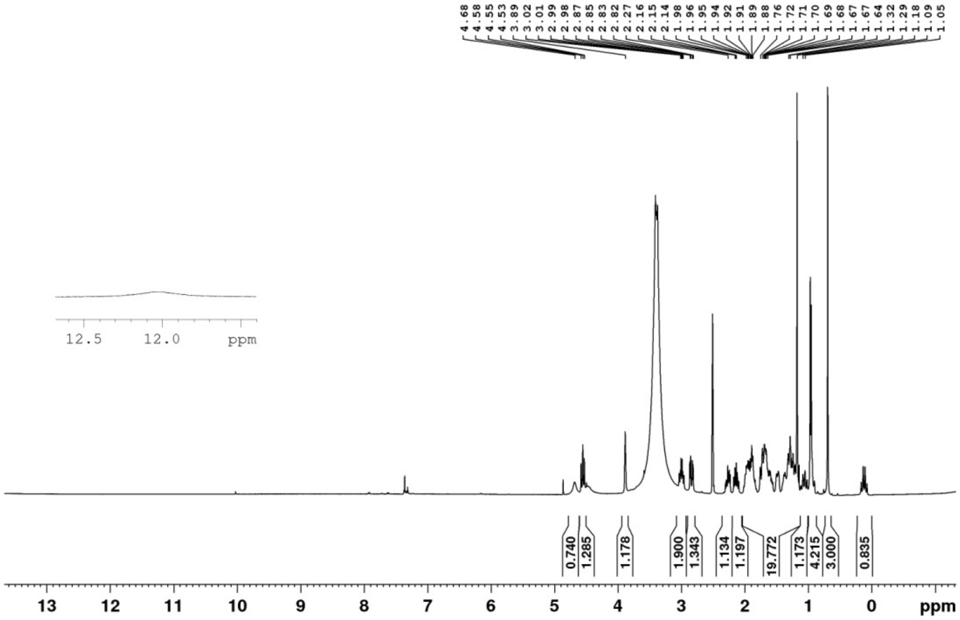

Compound 24, DMSO-d6, 101 MHz

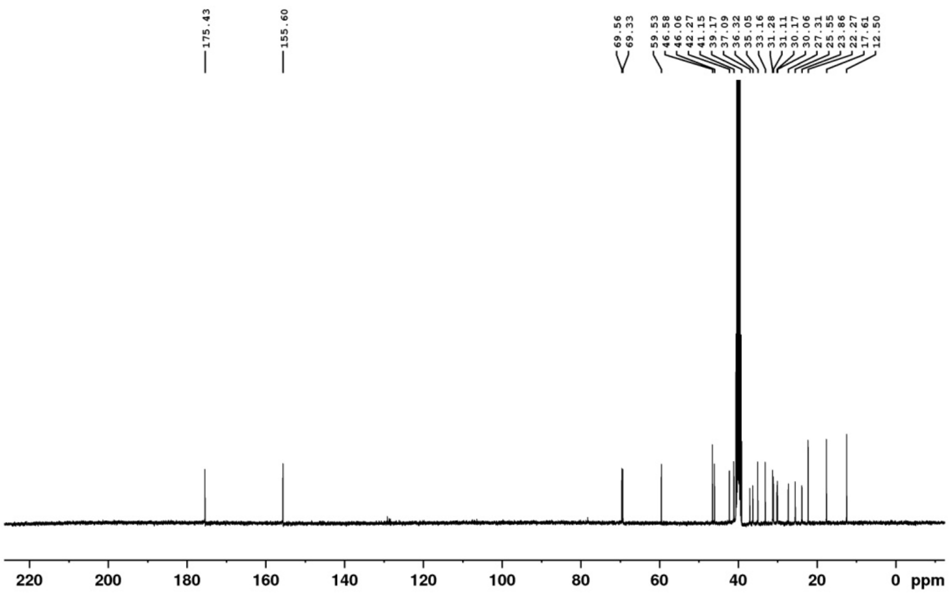

5. References

1. G. García Liñares, M. Antonela Zígolo, L. Simonetti, S. A. Longhi, A. Baldessari, *Enzymatic synthesis of bile acid derivatives and biological evaluation against Trypanosoma cruzi,* Bioorg. Med. Chem. 23 (2015) 4804–4814
2. J. Moural , K. Syhora, *Reaction of azoimide with steroidal 4-en-3-ones,* Collection Czechoslov. Chem. Commun. 35 (1970) 2018-2029.
3. Rigaku OD (2015). CrysAlis PRO. Rigaku Oxford Diffraction Ltd, Yarnton, Oxfordshire, England. 2015.
4. R.H. Blessing, *An empirical correction for absorption anisotropy.* Acta Cryst. A 51 (1995) 33–38, DOI: 10.1107/S0108767394005726
5. G.M. Sheldrick, *SHELXT – Integrated Space-Group and Crystal-Structure Determination.* Acta Crystallogr. Sect. A Found. Adv. 71 (2015) 3–8, DOI: 10.1107/S2053273314026370
6. G.M. Sheldrick, *Crystal Structure Refinement with SHELXL.* Acta Crystallogr. Sect. C Struct. Chem. 71 (2015) 3–8, DOI: 10.1107/S2053229614024218
